# H3K27me3 Profiling of the Endosperm Implies Exclusion of Polycomb Group Protein Targeting by DNA Methylation

**DOI:** 10.1371/journal.pgen.1001152

**Published:** 2010-10-07

**Authors:** Isabelle Weinhofer, Elisabeth Hehenberger, Pawel Roszak, Lars Hennig, Claudia Köhler

**Affiliations:** 1 Department of Biology and Zurich-Basel Plant Science Center, Swiss Federal Institute of Technology, Zurich, Switzerland; 2 Department of Plant Biology and Forest Genetics, Uppsala BioCenter, Swedish University of Agricultural Sciences, Uppsala, Sweden; National Institute of Genetics, Japan

## Abstract

Polycomb group (PcG) proteins act as evolutionary conserved epigenetic mediators of cell identity because they repress transcriptional programs that are not required at particular developmental stages. Each tissue is likely to have a specific epigenetic profile, which acts as a blueprint for its developmental fate. A hallmark for Polycomb Repressive Complex 2 (PRC2) activity is trimethylated lysine 27 on histone H3 (H3K27me3). In plants, there are distinct PRC2 complexes for vegetative and reproductive development, and it was unknown so far whether these complexes have target gene specificity. The FERTILIZATION INDEPENDENT SEED (FIS) PRC2 complex is specifically expressed in the endosperm and is required for its development; loss of FIS function causes endosperm hyperproliferation and seed abortion. The endosperm nourishes the embryo, similar to the physiological function of the placenta in mammals. We established the endosperm H3K27me3 profile and identified specific target genes of the FIS complex with functional roles in endosperm cellularization and chromatin architecture, implicating that distinct PRC2 complexes have a subset of specific target genes. Importantly, our study revealed that selected transposable elements and protein coding genes are specifically targeted by the FIS PcG complex in the endosperm, whereas these elements and genes are densely marked by DNA methylation in vegetative tissues, suggesting that DNA methylation prevents targeting by PcG proteins in vegetative tissues.

## Author Summary

Cell identity is established by the evolutionary conserved Polycomb group (PcG) proteins that repress transcriptional programs which are not required at particular developmental stages. The plant FERTILIZATION INDEPENDENT SEED (FIS) PcG complex is specifically expressed in the endosperm where it is essential for normal development. The endosperm nourishes the embryo, similar to the physiological function of the placenta in mammals. In this study, we established the cell type–specific epigenome profile of PcG activity in the endosperm. The endosperm has reduced levels of DNA methylation, and based on our data we propose that PcG proteins are specifically targeted to hypomethylated sequences in the endosperm. Among these endosperm-specific PcG targets are genes with functional roles in endosperm cellularization and chromatin architecture, implicating a fundamental role of PcG proteins in regulating endosperm development. Importantly, we identified transposable elements and genes among the specific PcG targets in the endosperm that are densely marked by DNA methylation in vegetative tissues, suggesting an antagonistic placement of DNA methylation and H3K27me3 at defined sequences.

## Introduction

Polycomb group (PcG) proteins are evolutionary conserved master regulators of cell identity and balance the decision between cell proliferation and cell differentiation [1]. PcG proteins act in multimeric complexes that repress transcription of target genes; the best characterized complexes are the evolutionary conserved Polycomb Repressive Complex 2 (PRC2) that catalyzes the trimethylation of histone H3 on lysine 27 (H3K27me3), and PRC1, which binds to this mark and catalyzes ubiquitination of histone H2A at lysine 119 [1]. Plants contain multiple genes encoding homologs of PRC2 subunits that have different roles during vegetative and reproductive plant development [2]. Whereas the EMBRYONIC FLOWER (EMF) and VERNALIZATION (VRN) complexes control vegetative plant development, reproductive development in Arabidopsis crucially depends on the presence of the FERTILIZATION INDEPENDENT SEED (FIS) PcG complex that is comprised of the subunits MEDEA (MEA), FERTILIZATION INDEPENDENT SEED2 (FIS2), FERTILIZATION INDEPENDENT ENDOSPERM (FIE) and MSI1 [2]. The FIS PcG complex is required to suppress autonomous endosperm development; loss of FIS function initiates the fertilization-independent formation of seed-like structures containing diploid endosperm [3]. In most angiosperms the endosperm is a triploid zygotic tissue that develops after fusion of the homodiploid central cell with a haploid sperm cell. The endosperm regulates nutrient transfer to the developing embryo and regular endosperm development is essential for embryo development [4]. Loss of FIS function also dramatically impacts on endosperm development after fertilization, causing endosperm overproliferation and cellularization failure, eventually leading to seed abortion [5]. Thus far, only few direct target genes of the FIS PcG complex are known, among them the MADS-box transcription factor *PHERES1* (*PHE1*) [6], *FUSCA3* [7] and *MEA* itself [8]–[10]. All three genes are also targets of vegetatively active PcG complexes [7], [11], suggesting that different PcG complexes share at least a subset of target genes [7].

Similar to extraembryonic tissues in mammals [12], the endosperm has reduced levels of DNA methylation compared to the embryo or vegetative tissues [13], [14]. Hypomethylation is established by transcriptional repression of the maintenance DNA-methyltransferase *MET1* during female gametogenesis [15], together with active DNA demethylation by the DNA glycosylase DEMETER (DME) [13], [16]. Whereas the global DNA methylation levels differ only slightly between embryo and endosperm (∼6% for CG methylation), methylation differences at transposable elements and repeat sequences are significantly more pronounced [13], [14]. The functional significance of this genome-wide demethylation of the endosperm is not yet understood. However, it has been proposed that DNA demethylation might cause transposon activation and generation of small interfering RNAs (siRNA) that might move to egg cell or embryo where siRNA-mediated DNA methylation would lead to increased methylation of parasitic genomic sequences [13]. This notion is supported by the observation of accumulating 24nt siRNAs in the female gametophyte and in the endosperm [17]. However, functional loss of RNA polymerase IV, the enzyme responsible for the biogenesis of siRNAs, does not cause reactivation of most transposons [18], suggesting the presence of redundant pathways to silence transposable elements.

In this study, we profiled the H3K27me3 pattern in the endosperm and identified many target genes that were known previously to be targeted by vegetatively active PcG complexes, supporting the idea that different PcG complexes share a common set of target genes. However, we also identified endosperm-specific H3K27me3 target genes that have functional roles in endosperm cellularization and chromatin architecture, suggesting that the FIS PcG complex has endosperm-specific functions and that PcG targeting in plants has tissue specific roles. Finally and most importantly, we discovered that the FIS PcG complex in the endosperm targets transposable elements (TEs) that are protected by DNA methylation in vegetative tissues, implicating that DNA methylation and H3K27me3 are alternative repressive marks that may compensate for each other in the repression of a subset of TEs.

## Results

### Isolation of Endosperm Nuclei by Fluorescent Activated Cell Sorting

**Figure 1:**
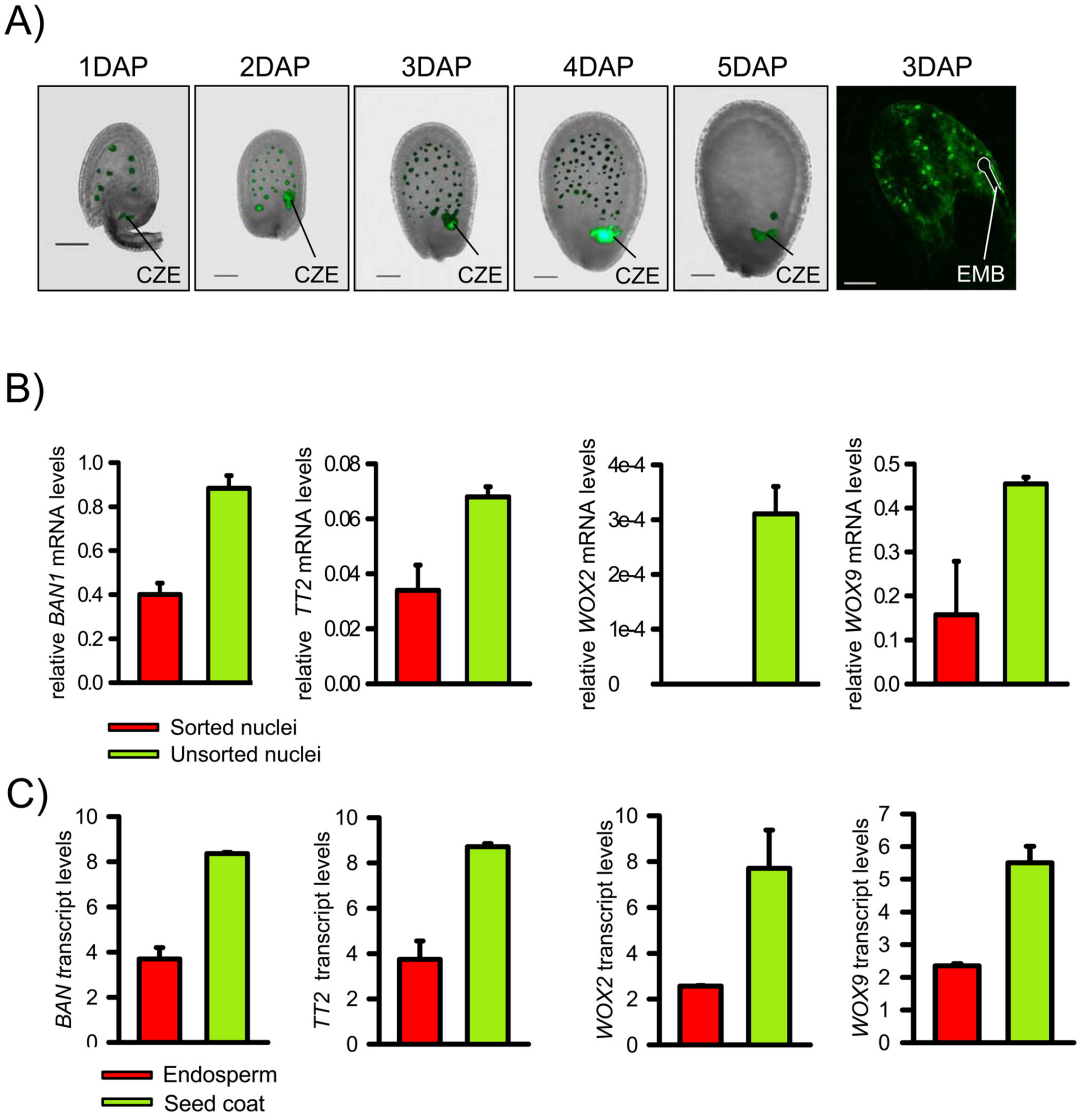
Isolation of EGFP Positive Endosperm Nuclei and Validation of the Technique. A) *PHE1::PHE1-EGFP* is specifically expressed in endosperm nuclei from 1 DAP to 4 DAP. First five images are fluorescence images overlaid with bright-field images. The chalazal endosperm (CZE) is indicated. Last image of the row shows a confocal image. The position of the embryo (EMB) is indicated by a white line. Scale bars, 50 µM. B) Quantitative RT-PCR expression analysis of seed coat marker genes *BANYULS* (*BAN*) and *TRANSPARENT TESTA2* (*TT2*), and embryo marker genes *WUSCHEL-RELATED HOMEOBOX* (*WOX*) *2* and *WOX9* in sorted endosperm nuclei and total nuclei isolated from *PHE1::PHE1-EGFP* expressing 1–4 DAP-old seeds. Values are shown relative to *PHE1* expression. Error bars, s.e.m. C) Transcript levels of seed coat marker genes *BAN* and *TT2* and embryo marker genes *WOX2* and *WOX9* in peripheral endosperm and seed coats of seeds from 1 DAP to 3 DAP, corresponding to seeds containing preglobular to globular stage embryos. Values are based on ATH1 microarray signals after RMA normalization. Error bars, s.e.m.

**Figure 2:**
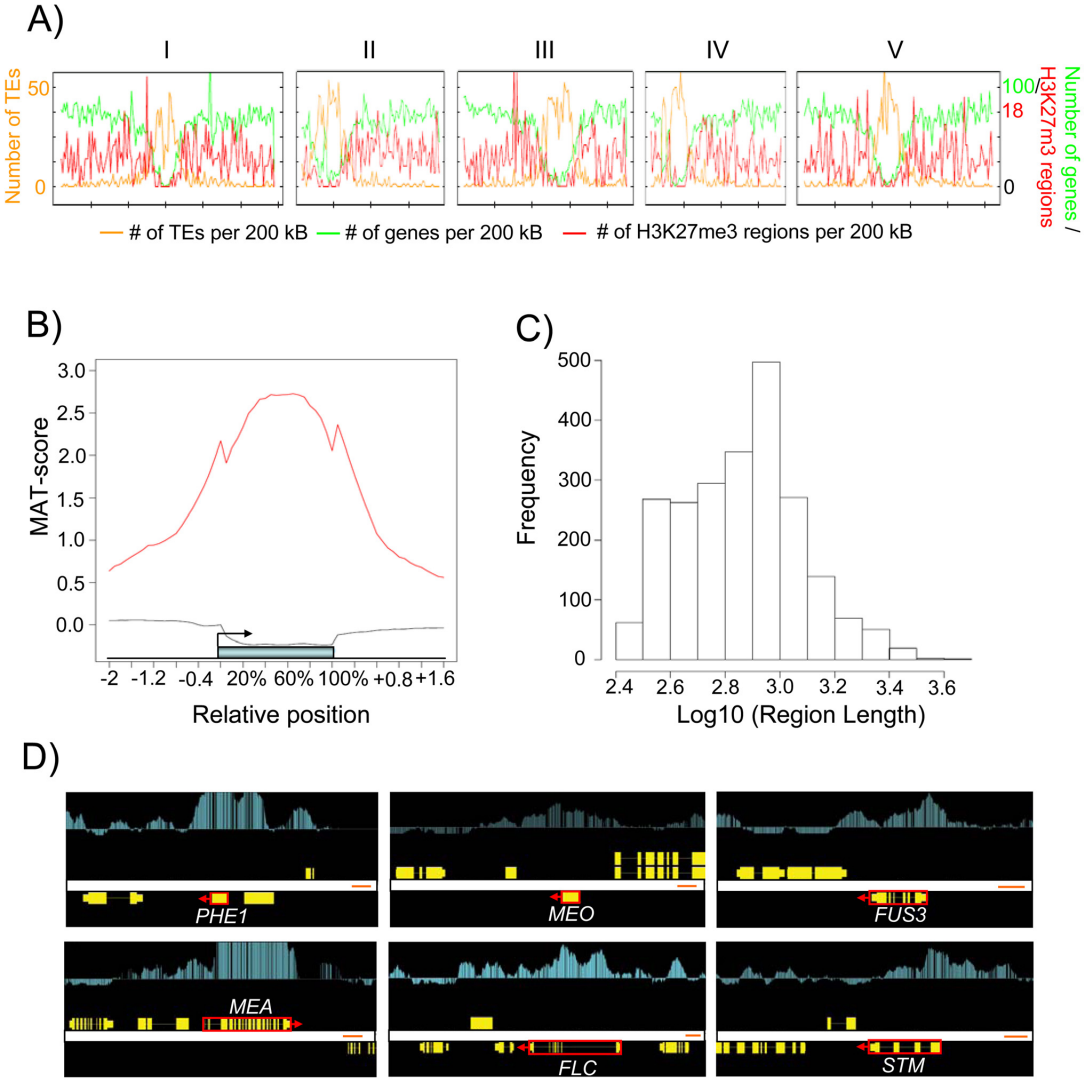
Genome-Wide Identification of H3K27me3 Regions in the Endosperm. A) Chromosomal distribution of H3K27me3 regions. The H3K27me3 regions per 200 kb and genes per 200 kb (y-axis, right-side scale) and number of transposons (y-axis, left-side scale). Numbers on top indicate chromosome number. B) Average H3K27me3 profiles (red line) over H3K27me3 targets. The black line represents the H3K27me3 profile over genes not marked by H3K27me3. The blue bar represents the annotated gene body from transcription start (left) to transcription end (right). Profiles are shown for 5% length intervals along the gene body and for 100 bp sequence intervals for the 2-kb regions upstream and downstream of each gene. C) Length distribution of H3K27me3 regions. D) Comparison of ChIP-chip results with Arabidopsis genes (red boxes, where arrows indicate direction of transcription) that were previously shown to be H3K27me3 targets [6], [7], [11], [57]. Genes are shown as yellow boxes (exons) and lines (introns), and H3K27me3 is shown as vertical light blue bars [MAT score ranging from −1 (bottom) to 6 (top)].

**Figure 3:**
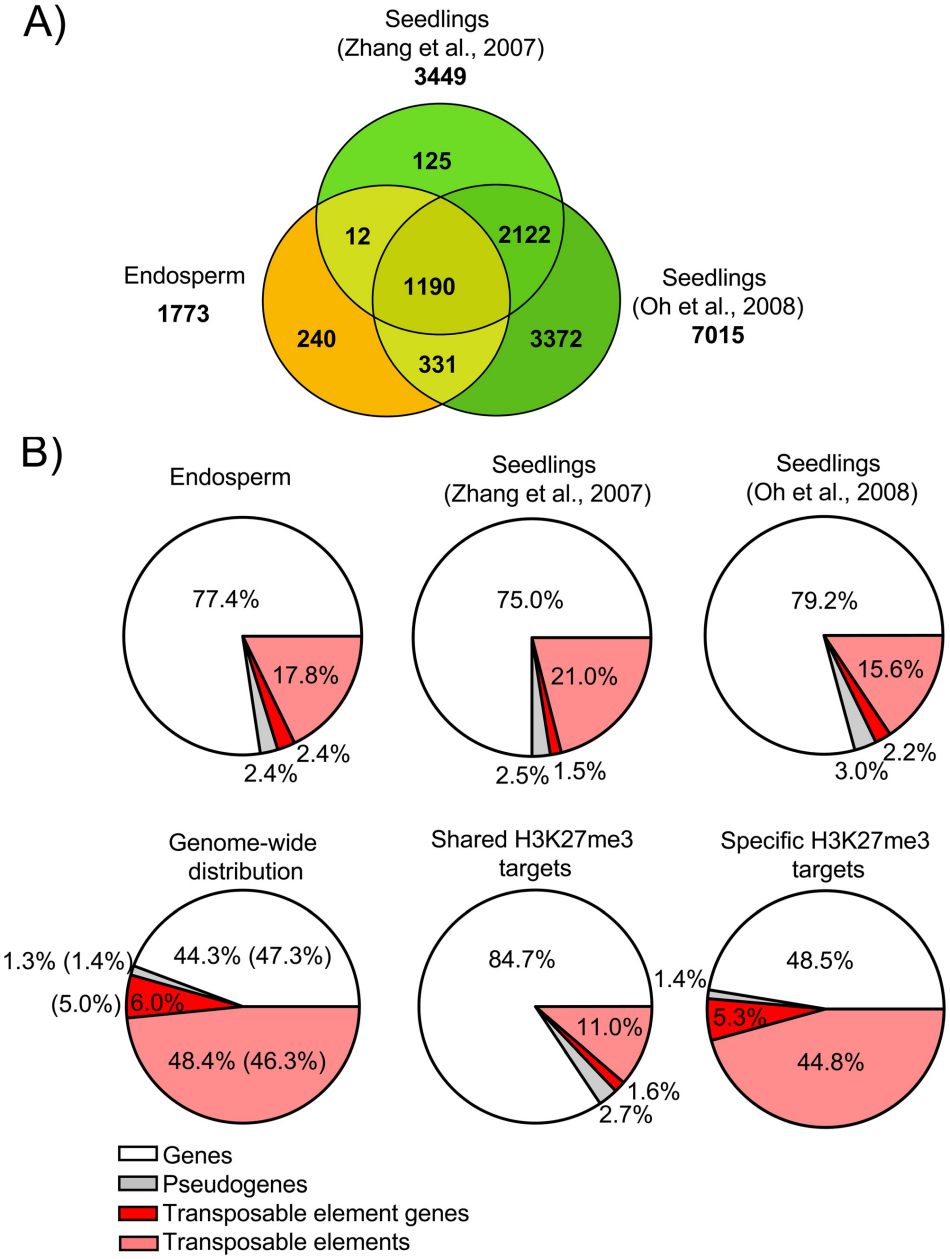
Characteristics of H3K27me3 Target Genes in the Endosperm. A) Venn diagram showing overlap of H3K27me3 target genes in seedlings [11], [19] and endosperm. B) Distribution of different types of H3K27me3 targets (genes, pseudogenes, transposable elements, transposable element genes) in endosperm and seedling tissues. Upper panel shows distribution in the endosperm (this study) and seedlings [11], [19], lower panel shows genome-wide distribution of genes, pseudogenes, transposable elements and transposable element genes in comparison to the distribution of shared and specific endosperm H3K27me3 targets. Number in parenthesis reflect number of detectable targets.

We established a transgenic line expressing PHE1 fused to the enhanced green fluorescent protein (EGFP) under control of the native promoter and 3′ regulatory elements. Strong EGFP fluorescence was exclusively detected in endosperm nuclei from 1 day after pollination (DAP) until 4 DAP, whereas only a weak signal was detectable in the chalazal endosperm at 5 DAP (Figure 1A). EGFP-labeled nuclei from 1–4 DAP-old seeds were isolated with the use of a fluorescence-activated cell sorter. High-throughput techniques allowed the harvesting, nuclei isolation, and sorting of approximately 100 000 nuclei in about 4 hours. Within this time period, endosperm nuclei did apparently not undergo substantial changes in their transcriptional identity, as judged by a relatively low expression of embryo and seed coat marker genes in relation to the *PHE1* gene (Figure 1B). Expression of seed coat and embryo marker genes followed a similar trend in microdissected endosperm samples (Figure 1C). To identify endosperm-specific PcG target genes we performed chromatin immunoprecipitation (ChIP) of chromatin from sorted endosperm nuclei using H3K27me3 specific antibodies followed by hybridization to high resolution whole-genome tiling microarrays (Chip-on-chip). As a control, we performed ChIP with unspecific IgG antibodies. Genomic regions marked by H3K27me3 (“H3K27me3 regions”) were identified as continuous runs of probes with a MAT-score of at least 3.5 (see Materials and Methods). We identified 2282 regions that were significantly enriched for H3K27me3, covering ∼1.9 Mb and representing ∼1.6% of the sequenced genome. This corresponds to about one fourth the number of H3K27me3 regions identified in seedling tissues [11], [19], indicating that there are substantially fewer H3K27me3 targets in the endosperm than in vegetative tissues. Similar to the H3K27me3 distribution in Arabidopsis seedlings [11], most H3K27me3 regions in the endosperm were located on euchromatic chromosome arms and only 17 of the 2282 regions (0.7%) were from centromeric or pericentromeric heterochromatin (Figure 2A). The distribution of H3K27me3 in endosperm over genes had a pronounced maximum in the transcribed region, similar to the distribution of H3K27me3 in vegetative tissues (Figure 2B, [11]). Notably, there was a small but distinct drop of H3K27me3 at the transcriptional start and shortly after the transcriptional stop, possibly caused by localized nucleosome depletion. This interpretation would be in agreement with previous observations made in yeast and human cells, revealing nucleosome depletion at the transcriptional start and around polyadenylation sites [20]–[22]. The length of H3K27me3 regions in the endosperm was comparable to the length of H3K27me3 regions in vegetative tissues [11], with a median region size of about 750 bps (Figure 2C). *MEA*, *PHE1*, *MEIDOS (MEO)* and *FUSCA3 (FUS3)* as well as other genes that were previously identified as sporophytic H3K27me3 targets were among the endosperm H3K27me3 targets (Figure 2D and Figure 3A), indicating that our procedure successfully identified H3K27me3 targets in the endosperm.

### Transposable Elements Are Specifically Targeted by H3K27me3 in the Endosperm

We identified 1773 genes to be associated with H3K27me3; of those, 1533 genes (∼86.5%) overlapped with H3K27me3 marked loci identified in seedling tissues (“shared H3K27me3 targets”) [11], [19], whereas 240 loci (∼13.5%) were specifically enriched only in the endosperm (“endosperm-specific H3K27me3 targets”) (Figure 3A and Table S1). Most H3K27me3 targets in both sample sets are protein-coding genes of known or unknown functions, similar to the H3K27me3 targets in seedling tissues [11], [19] (Figure 3B). The overall distribution of H3K27me3 marked pseudogenes and TEs in the endosperm and seedling tissues was similar; TEs and transposable element genes (TEGs; correspond to genes encoded within a transposable element) were clearly underrepresented among H3K27me3 targets compared to the genome average (Figure 3B). However, the frequency of TEs and TEGs was much higher among the endosperm-specific H3K27me3 targets than among the shared H3K27me3 targets, indicating that a subset of TEs and TEGs are specifically marked by H3K27me3 in the endosperm (Figure 3B). While 16% of all TEs and 46% of all TEGs probed by the microarray are located in centromeric and pericentromeric heterochromatin, only 5% of the TEs with H3K27me3 and 16% of the TEGs with H3K27me3 were from these heterochromatic regions. Frequencies of almost all super families of TEs were similar among H3K27me3-marked endosperm-specific TEs and among all TEs detectable by the microarray (Figure S1). Among the shared H3K27me3 targets LTR/COPIA (p<5E-4), LINE/L1 (p<0.05), and RathE1 elements (p<0.05) were significantly enriched, indicating non-random targeting of TEs by PcG proteins. We verified the specificity of our analysis by qPCR validation of endosperm-specific and shared H3K27me3 targets using independently prepared ChIP samples. We randomly selected 10 endosperm-specific TEGs, 9 endosperm-specific genes and 8 shared target genes and could confirm all loci in an independent ChIP experiment (Figure S2), indicating that our procedure was specific with a low false discovery rate.

### Functional Roles and Expression of H3K27me3 Target Genes in the Endosperm

Shared H3K27me3 targets in the endosperm were highly enriched for genes involved in transcriptional regulation, with MADS-box transcription factors being a prominently enriched subclass of transcription factors (p = 3.01E-05; Table S2). However, many other GO categories were enriched among shared H3K27m3 target genes, including regulation of metabolism, flower development, cell wall organization, secondary metabolism and others (Table S3). This indicates that the FIS PcG complex acts to repress a large set of genes that are not required during early endosperm development. Among endosperm-specific H3K27me3 targets, there were many genes with potential roles in vesicle-mediated transport and cytoskeleton organization (Table S4), suggesting a specific function of the FIS PcG complex in endosperm cellularization. Furthermore, many genes with functional roles in chromatin organization, such as the PcG protein encoding genes *EMF2*, *VRN2*, *MSI1*, the DNA glycosylase *ROS1* as well as DNA helicases were among specific H3K27me3 target genes (Table S4), implicating a role of the FIS PcG complex in establishing specific chromatin architectures in the endosperm.

**Figure 4:**
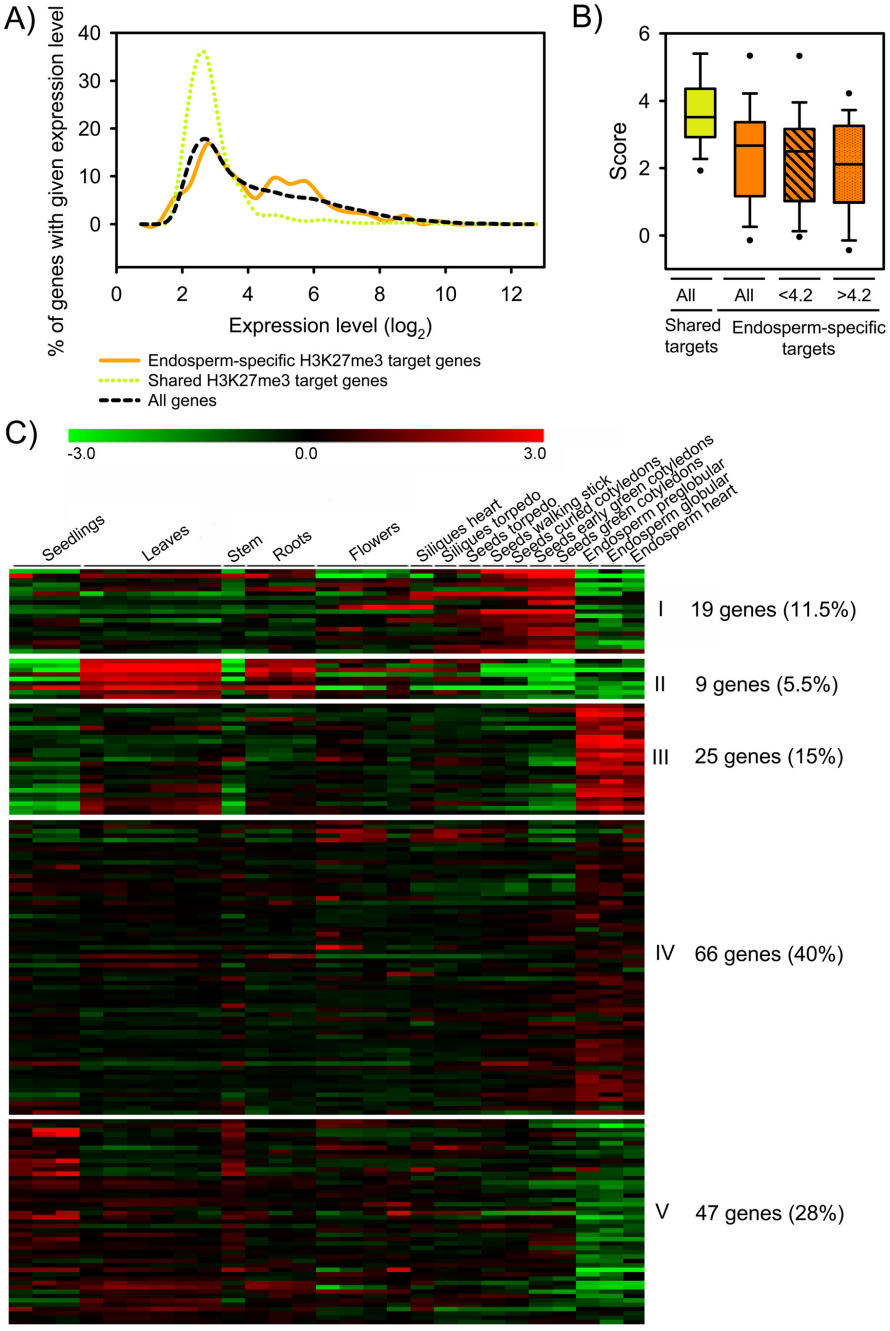
Expression of H3K27me3 Target Genes in the Endosperm. A) Expression level of shared (green) and endosperm-specific (orange) H3K27me3 target genes compared to all genes (black) in the peripheral endosperm of seeds containing globular stage embryos. B) Box plot of H3K27me3 MAT scores of shared (green) and endosperm-specific (orange) target genes. H3K27me3 MAT scores of endosperm-specific target genes with low expression levels (log_2_<4.2) and moderate expression levels in the endosperm (log_2_>4.2) are symbolized by striped and dotted fill patterns, respectively. C) Cluster analysis of endosperm-specific H3K27me3 target genes. H3K27me3 target genes are grouped into five mutually exclusive clusters based on their expression patterns. Each row represents a gene, and each column represents a tissue type. Tissue types are: seedlings, leaves, stems, roots, flowers, siliques containing seeds with embryos in the heart or torpedo stage, seeds with embryos in the torpedo, walking stick, curled cotyledon, early green, and green cotyledon stage and endosperm derived from seeds with embryos in the preglobular, globular and heart stage. Red or green indicate tissues in which a particular gene is highly expressed or repressed, respectively.

Next, we analyzed the relation between H3K27me3 modification and gene expression. Gene expression data were derived from the peripheral endosperm of seeds containing globular stage embryos, corresponding to the main fraction of the sorted endosperm nuclei used in our ChIP-chip experiment. Consistent with the function of H3K27me3 in transcriptional silencing, the majority of shared endosperm H3K27me3 target genes were expressed at low levels (Figure 4A). In contrast, a fraction of the endosperm-specific H3K27me3 targets was moderately expressed (Figure 4A). Endosperm-specific H3K27me3 target genes had lower average H3K27me3 scores compared to shared targets independent of their expression level (Figure 4B), suggesting that there is different efficiency of PcG protein targeting or PRC2 activity for endosperm-specific versus shared endosperm H3K27me3 targets.

Using publicly available datasets we tested the tissue-specific expression of endosperm-specific H3K27me3 target genes by cluster analysis. Consistent with the idea that the FIS PcG complex is required for repression of target genes in the endosperm, genes present in clusters I, II and V (45%, n = 75) were specifically repressed in the endosperm (Figure 4C). However, about half of all endosperm-specific H3K27me3 targets were expressed in the endosperm (clusters III and IV, 55%, n = 91; Figure 4C), in agreement with the higher average expression levels of endosperm-specific H3K27me3 target genes compared to non-H3K27me3 target genes (Figure 4A). We consider three not mutually exclusive explanations for this observation: (i) H3K27me3 is not necessarily connected with gene silencing in the endosperm. (ii) For a subset of genes only one of the alleles is marked by H3K27me3. In this case expression of the non-marked allele would be detected, whereas the H3K27me3 allele remains silenced, as it was shown before for *PHE1* and *MEA* [8], [9], [23], [24]. However, imprinted genes predicted by Gehring and colleagues [14] were not among genes present in clusters III and IV. (iii) PcG target genes are differentially regulated in the different domains of the endosperm, i.e. the micropylar, peripheral and chalazal domains).

### DNA Methylated Loci Become Targets of H3K27me3 in the Endosperm

**Figure 5:**
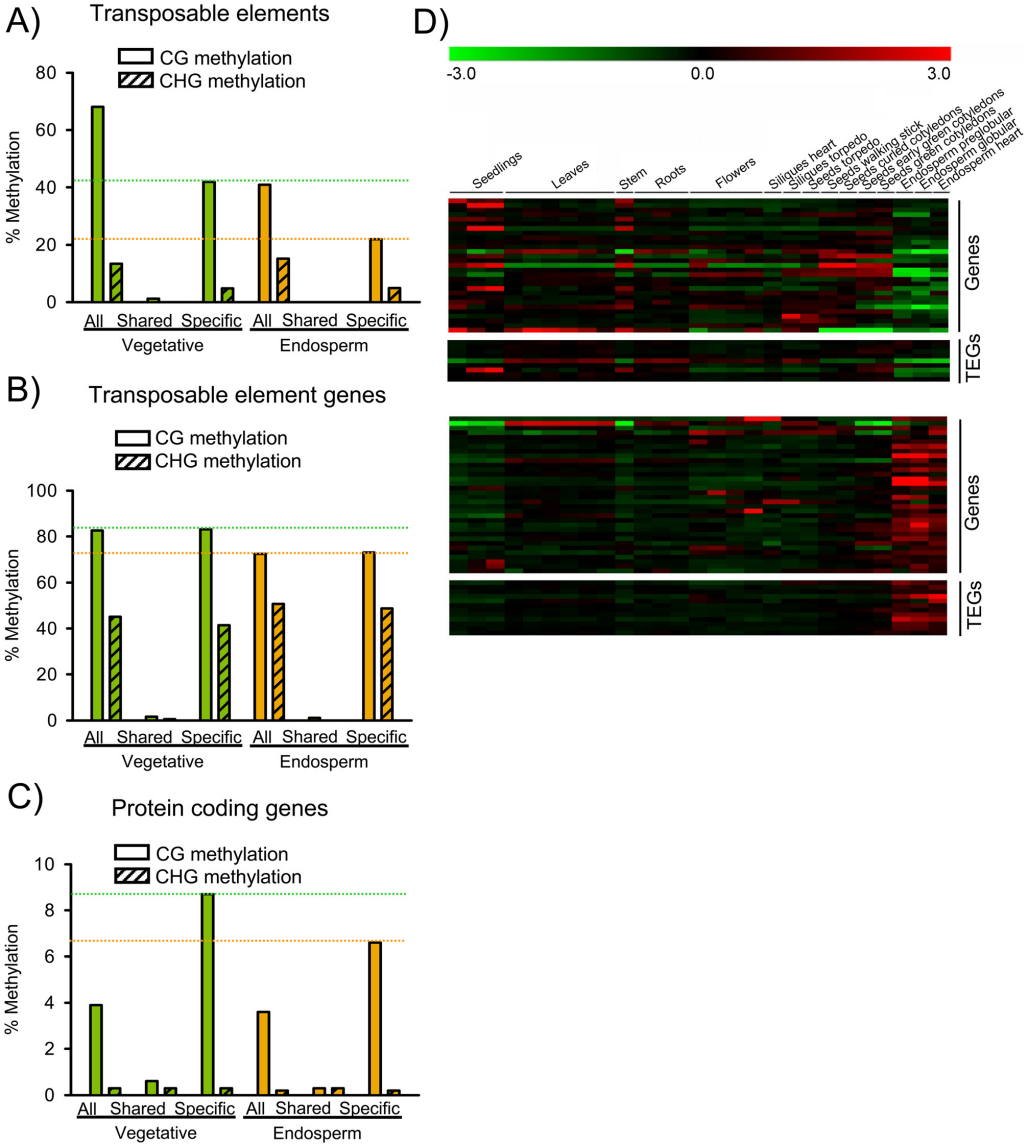
DNA Methylated Genes Become Targets for H3K27me3 in the Endosperm. A) Median DNA methylation levels of TEs in vegetative tissues (green) and endosperm (orange). CG and CHG methylation levels were analyzed for all TEs, TEs marked specifically by H3K27me3 in the endosperm (“Specific”) and TEs marked by H3K27me3 in the endosperm and in seedlings [11], [19] (“Shared”). Median DNA methylation levels of shared TEs are close to zero. Green and orange dotted horizontal lines mark CG methylation levels of specific H3K27me3 TEs in vegetative and endosperm tissues, respectively. B) Median DNA methylation levels of TEGs in vegetative tissues (green) and endosperm (orange). CG and CHG methylation levels were analyzed for all TEGs, TEGs marked specifically by H3K27me3 in the endosperm (“Specific”) and TEGs marked by H3K27me3 in the endosperm and in seedlings [11], [19] (“Shared”). Median DNA methylation levels of shared TEGs are close to zero. Green and orange dotted horizontal lines mark CG methylation levels of specific H3K27me3 TEGs in vegetative and endosperm tissues, respectively. C) Median DNA methylation levels of protein coding genes in vegetative tissues (green) and endosperm (orange). CG and CHG methylation levels were analyzed for all protein coding genes, protein coding genes marked specifically by H3K27me3 in the endosperm (“Specific”) and protein coding genes marked by H3K27me3 in the endosperm and in seedlings [11], [19] (“Shared”). Green and orange dotted horizontal lines mark CG methylation levels of specific H3K27me3 protein coding genes in vegetative and endosperm tissues, respectively. D) Cluster analysis of DNA methylated H3K27me3 target genes and transposons. Genes and transposons are grouped into two mutually exclusive clusters based on their expression patterns in different tissues. Each row represents a gene, and each column represents a tissue type. Tissue types are: seedlings, leaves, stems, roots, flowers, siliques containing seeds with embryos in the heart or torpedo stage, seeds with embryos in the torpedo, walking stick, curled cotyledon, early green, and green cotyledon stage and endosperm derived from seeds with embryos in the preglobular, globular and heart stage. Red or green indicate tissues in which a particular gene is highly expressed or repressed, respectively.

TEs were strongly overrepresented among the endosperm-specific H3K27me3 targets compared to the shared H3K27me3 targets (Figure 3B). Hence, we hypothesized that the global DNA demethylation in the endosperm [13], [14] caused H3K27me3 to accumulate in regions that are DNA methylated in vegetative tissues and, therefore, H3K27me3-poor. This hypothesis predicts that TEs marked by H3K27me3 in the endosperm have reduced endosperm DNA methylation levels compared to all TEs. Indeed, median endosperm CG and CHG DNA methylation levels were lower at H3K27me3 marked TEs than at other TEs (Figure 5A). CHH methylation levels were generally low and did not differ between H3K27me3 marked TEs and all TEs (data not shown). TEs that carried H3K27me3 in endosperm and vegetative tissues were almost devoid of CG DNA methylation in endosperm and vegetative tissues. In contrast, TEs that carried H3K27me3 only in the endosperm had high DNA methylation levels in vegetative tissues while DNA methylation levels in the endosperm were markedly below the average over all TEs. Similarly, shared TEGs were almost devoid of DNA methylation in vegetative tissues and in the endosperm. Endosperm DNA methylation levels of specific H3K27me3 TEGs were comparable to the average DNA methylation levels in the endosperm of all TEGs present in the genome (Figure 5B), indicating that reduced DNA methylation levels in the endosperm might allow targeting of PcG proteins to defined sequences independent of residual DNA methylation. CHG methylation followed a similar trend as CG methylation (Figure 5B). In contrast, no substantial changes in CHH methylation levels were observed (data not shown). Protein coding genes were generally much less DNA methylated than TEs or TEGs. Similar to shared TEs and TEGs, shared H3K27me3 target genes were almost devoid of DNA methylation in vegetative tissues and the endosperm (Figure 5C). In marked contrast, endosperm-specific H3K27me3 target genes had significantly higher CG DNA methylation levels in vegetative tissues than the genome-wide average (Figure 5C), supporting the idea that CG DNA methylation prevents these genes being targeted by PcG proteins in vegetative tissues. CG DNA methylation level of endosperm-specific H3K27me3 genes was reduced in the endosperm compared to vegetative tissues, again suggesting that reduced DNA methylation levels in the endosperm enable targeting of PcG proteins to selected loci. Shared and specific protein coding H3K27me3 target genes were almost devoid of CHG and CHH methylation in vegetative tissues and the endosperm (Figure 5C and data not shown). Together, we conclude that DNA methylation and H3K27me3, which both can bring about transcriptional repression of target genes, usually exclude each other at target chromatin. In the endosperm, where DNA methylation is naturally reduced, some loci that were DNA methylated in other tissues become targeted by the FIS PcG complex and marked by H3K27me3. This hypothesis predicts that experimental reduction of DNA methylation levels in vegetative tissues will cause PcG proteins to be targeted to some loci that are usually DNA methylated. Indeed, in *met1* mutants H3K27me3 was found at some TEs that did not carry H3K27me3 in wild type [25], strongly supporting this idea.

Based on their expression in the endosperm, two main clusters of protein coding genes and TEGs that were DNA methylated in vegetative tissues and carried H3K27me3 in the endosperm were apparent (Figure 5D); the first cluster contained genes and TEGs that were weakly expressed in other tissues and became specifically repressed in the endosperm, whereas the second cluster contained genes and TEGs that were mainly repressed in other tissues and became specifically expressed in the endosperm, indicating that loss of DNA methylation fostered expression of several genes and transposons in the endosperm independent of their gain of H3K27me3.

### Only Few H3K27me3 Target Genes Are Deregulated in *fis2* Mutants

**Figure 6:**
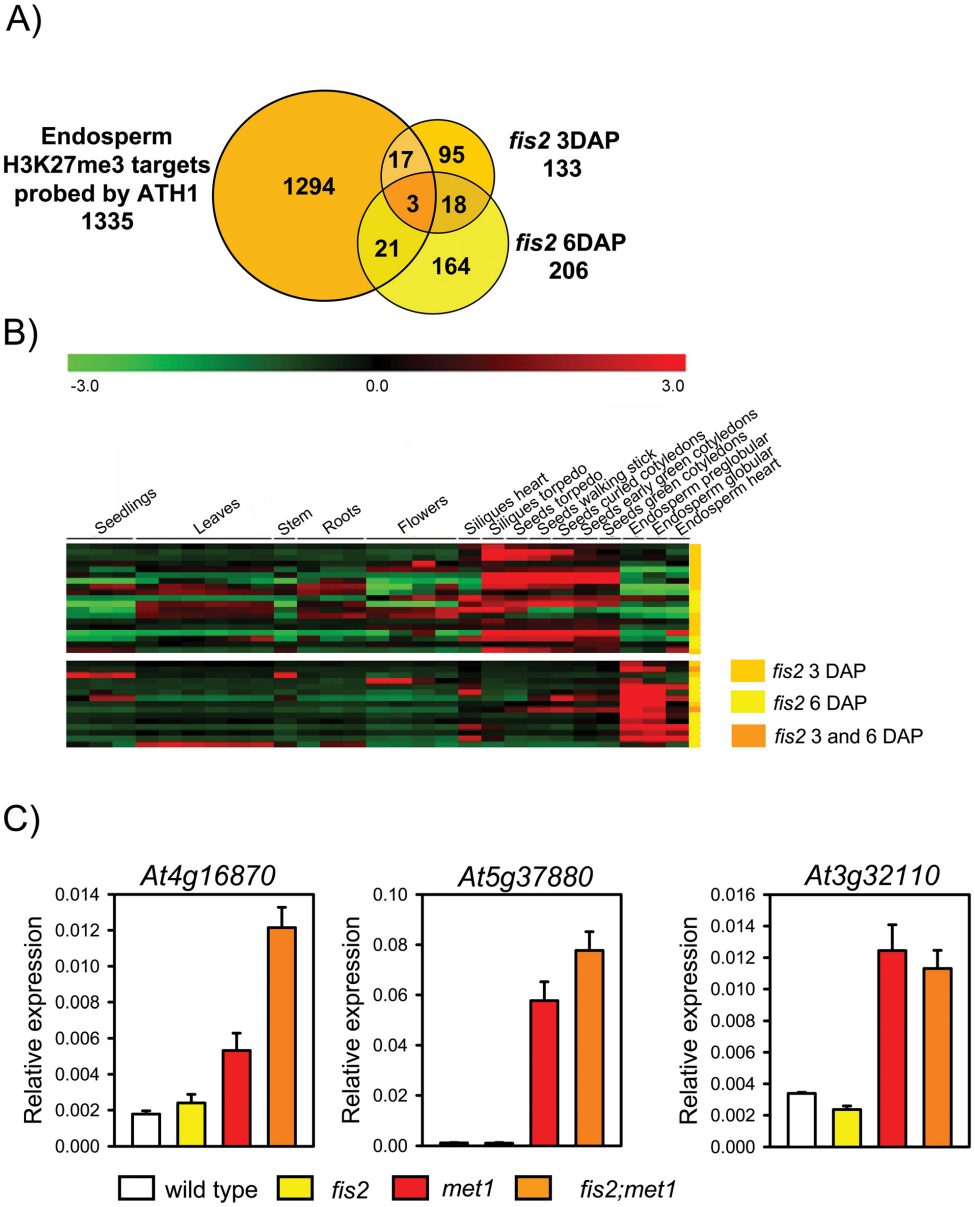
Only Few H3K27me3 Target Genes Are Deregulated in *fis2* Mutants. A) Venn diagram showing overlap of H3K27me3 target genes with genes deregulated in *fis2* seeds at 3 DAP and 6 DAP. Only genes present on the ATH1 microarray were included in the analysis. B) Cluster analysis of H3K27me3 target genes that are deregulated in *fis2* mutant. H3K27me3 target genes are grouped into two main clusters based on their expression patterns in different domains of the endosperm. Each row represents a gene, and each column represents a tissue type. Tissue types are: seedlings, leaves, stems, roots, flowers, siliques containing seeds with embryos in the heart or torpedo stage, seeds with embryos in the torpedo, walking stick, curled cotyledon, early green, and green cotyledon stage and endosperm derived from seeds with embryos in the preglobular, globular and heart stage. Red or green indicate tissues in which a particular gene is highly expressed or repressed, respectively. Colors at the right side symbolize genes deregulated in *fis2* at 3 DAP (orange), 6DAP (yellow), or at both time points (yellow with orange stripes). C) Quantitative RT-PCR analysis of TEGs in wild-type, *fis2/FIS2*, *met1/MET1* and *fis2/FIS2;met1/MET1* seeds. Error bars, s.e.m.

We wondered whether loss of FIS activity would cause a global deregulation of H3K27me3 target genes. Therefore, we profiled the *fis2* transcriptome of seeds harvested at 3 DAP and 6 DAP and searched for deregulated genes that were marked by H3K27me3 in the endosperm. Loss of FIS function profiled at 3 DAP and 6 DAP resulted in different and largely non-overlapping gene expression profiles (Figure 6A). Although the overlap of H3K27me3 target genes and genes deregulated upon loss of FIS function was significant (p = 3.0E-05 and 5.7E-04 for 3 DAP and 6 DAP, respectively), expression of surprisingly few target genes (∼1.5% and ∼1.8% at 3 DAP and 6 DAP, respectively) was increased upon loss of FIS function (Figure 6A, Table S5). *EMF2* and *VRN2* expression was not increased in *fis2* seeds at 3 or 6 DAP, indicating that loss of FIS2 function is not compensated by increased expression of *FIS2* homologous genes. Genes deregulated at 3 DAP and 6 DAP fell into two largely distinct clusters. Whereas most of early deregulated genes were not expressed in the wild-type endosperm until heart stage, late deregulated genes were predominantly expressed during early wild-type endosperm development and became repressed around heart stage (Figure 6B), supporting the idea that the FIS PcG complex is required for the repression of a defined set of genes around endosperm cellularization [26], [27]. Genes deregulated in *fis2* at 3 DAP and 6 DAP were prominently enriched for glycosyl hydrolases (Table S6), with a strong enrichment of Family 17 of plant glycoside hydrolases at 6 DAP. Family 17 members preferentially hydrolyse the major component of endosperm cell walls, callose, [28], suggesting that repression of cell wall degrading enzymes is a requirement for successful endosperm cellularization. Conversely, this implicates that increased expression of these genes in *fis* mutants might contribute to the failure of *fis* mutant endosperm to undergo endosperm cellularization [29].

Importantly, we did not detect increased expression of TEGs in *fis2* mutants, suggesting that loss of H3K27me3 might be compensated by other repressive mechanisms. If so, we wondered whether in seeds lacking both, FIS activity and CG DNA methylation, repression of TEGs would be relieved. Therefore, we generated *fis2/FIS2; met1/MET1* double mutants that contain 12.5% seeds homozygous for *met1* and devoid of FIS activity. We randomly selected eight endosperm-specific H3K27me3 TEGs (At4g16870, At5g37880, At3g32110, At2g13890, At5g35710, At1g35480, At3g28400, At2g16010) that were DNA methylated in vegetative tissues and had decreased DNA methylation levels in the endosperm (Figure S3). Among those, At4g16870, At5g37880 had increased expression levels in *fis2;met1* double mutants compared to *met1* and *fis2* single mutants (Figure 6C), whereas expression of At3g32110 equally increased in *met1* and *fis2; met1* double mutants. Expression of the other TEGs was not significantly changed compared to wild type (data not shown). Based on these data we conclude that DNA methylation and FIS-mediated H3K27me3 can act synergistically to repress a subset of TEGs in the endosperm, but that there are additional mechanisms to silence TEGs in the absence of both mechanisms.

## Discussion

Identification of tissue-specific target genes and unraveling how PcG proteins regulate their target genes are important steps to understand how tissue specificity is established. In this study we established the endosperm-specific H3K27me3 profile and the following main conclusions can be drawn based on our results: (1) The majority of PcG target genes are shared among the endosperm and vegetative tissues, indicating that the reproductively active FIS PcG complex and vegetatively active PcG complexes are recruited to a common set of genes. (2) Expression of only few PcG target genes is induced upon loss of FIS activity, suggesting the activation of alternative repressive mechanisms in the absence of PcG function and/or the lack of appropriate transcriptional activators in the endosperm. (3) Selected TEs, TEGs and protein coding genes are specifically targeted by the FIS PcG complex in the endosperm; these elements and genes are densely marked by DNA methylation in vegetative tissues, suggesting that DNA methylation prevents targeting by PcG proteins in vegetative tissues. (4) DNA demethylation in the endosperm may be required, but not sufficient for targeting of the FIS PcG complex. DNA demethylation in the endosperm is a global phenomenon [13], [14], whereas only selected loci become specifically targeted by the FIS PcG complex, suggesting that additional factors are decisive for PcG recruitment.

### Functional Roles of H3K27me3 Target Genes in the Endosperm

PcG proteins are largely viewed as general suppressors of genomic programmes that are not required in a specific tissue type or during a particular developmental stage of an organism [1]. This would predict that a large set of PcG target genes is shared in different tissues, as only a small set of genes is expressed in a tissue-specific fashion [30]. In line with this view, we found that the majority of PcG target genes identified in the endosperm are also targeted by PcG proteins in vegetative tissues [11], [19], suggesting that different PcG complexes share a common set of target genes during different stages of plant development. However, we identified substantially fewer PcG target genes in the endosperm than previous studies found in seedlings consisting of a mixture of many diverse cell types [11], [19] as well as in root hair and non-hair specific cell types [31].

The low number of identified H3K27me3 target genes in endosperm correlates well with reduced expression of the critical PRC2 components *MEA* and *FIS2* in the same tissue [8], [27]. A reason for lower expression of PcG proteins and only few PcG protein target genes in endosperm at 1–4 DAP could be that at this time, when mitotic activity is high, the endosperm has not yet acquired its terminal differentiation status [32]. In contrast, the cells profiled in the other studies [11], [19], [31] were mostly fully differentiated. This is similar to the situation in mammals, where lineage-specific genes often become targeted by PcG proteins only upon cell-fate commitment [33], leading to cell-type specific PcG target profiles and gene expression patterns [34], [35]. Furthermore, it should be noted that the endosperm has fundamentally different developmental origin and fate than vegetative tissues; it is derived after fertilization of the diploid central cell and will not contribute any cells to embryo and the developing new plant. Therefore, it is also possible that the reduced number of H3K27me3 target genes in the endosperm might reflect a less stringent requirement of PcG-mediated gene regulation in the endosperm than in vegetative tissues.

In the endosperm as well as in vegetative tissues, genes encoding for transcription factors were highly enriched among PcG target genes (this study and [11]), supporting the general idea that PcG proteins regulate cell identity by controlling expression of transcription factors [36]. Importantly however, H3K27me3 target genes were also prominently enriched for pectinesterases and glycosyl hydrolases - two enzyme classes that degrade major components of plant cell walls [28], [37], indicating an important role of the FIS PcG complex in the regulation of endosperm cellularization. The observed deregulation of both enzyme classes in *fis2* mutant seeds might be the underlying cause of endosperm cellularization failure of *fis* mutants [29].

### Only Few H3K27me3 Target Genes Are Deregulated upon Depletion of FIS Activity

Loss of FIS function caused deregulation of only few H3K27me3 genes, similar to observations made in mammalian and *Drosophila* cells, where only a small subset of PcG target genes were deregulated upon depletion of PcG proteins [33], [38], [39]. Stable repression of FIS target genes could be due to secondary epigenetic modifications that together with FIS-mediated H3K27me3 keep PcG target genes repressed and which are not alleviated in FIS-depleted cells. Alternatively, it is possible that secondary epigenetic modifications are only recruited to FIS target genes upon loss of FIS function. As a third and complementary explanation for the lack of expression of a large number of FIS target genes in FIS-depleted endosperm we propose that the promoters of many PcG target genes lack binding sites for endosperm-specific transcriptional activators required for substantially increased expression in this tissue. This last explanation would imply that those FIS target genes that are deregulated in the *fis2* mutant are even in wild type expressed in the endosperm. Indeed, deregulated FIS target genes were predominantly expressed during wild-type seed development (Figure 6B), supporting the hypothesis that cis-acting tissue-specific enhancers are required for full induction of FIS target genes upon loss of H3K27me3.

### Transposable Elements Are Targeted by the FIS PcG Complex in the Endosperm

TEs and TEGs were most prominently enriched among endosperm-specific H3K27me3 targets. This is in contrast to the situation in vegetative tissues, where these elements are largely excluded from PcG target genes [11]. We propose that reduced levels of DNA methylation in the endosperm allow targeting of the FIS PcG complex to defined sequence elements that are protected by DNA methylation in vegetative tissues. This conclusion is supported by the following findings made in this study: (i) Shared H3K27me3 targets were completely devoid of DNA methylation, indicating that DNA methylation prevents targeting by PcG proteins. (ii) Endosperm-specific H3K27me3 protein coding genes had much higher CG DNA methylation levels in vegetative tissues compared to genome-wide average DNA methylation levels, supporting the view that DNA methylation prevents these genes being targeted by PcG proteins in vegetative tissues. (iii) In the endosperm, the average DNA methylation level of endosperm-specific H3K27me3 targets was reduced compared to vegetative tissues. This trend was most pronounced for TEs, where DNA methylation level of endosperm-specific TEs were much lower compared to the genome-wide average DNA methylation of TEs in the endosperm. However, also TEGs and protein-coding genes had reduced DNA methylation levels in the endosperm compared to vegetative tissues, supporting the notion that reduced DNA methylation levels in the endosperm allow targeting of the FIS PcG complex to defined sequence elements. However, DNA demethylation is a global phenomenon [13], [14], but only selected sequences were targeted by the FIS complex, suggesting that DNA demethylation is necessary, but not sufficient for targeting of the FIS complex. The conclusion that DNA methylation and H3K27me3 are usually exclusive epigenetic marks is strongly supported by previous studies on seedlings with experimentally altered DNA methylation. When DNA methylation was reduced, H3K27me3 localized to defined regions within heterochromatin [25], and when DNA methylation was increased H3K27me3 levels dropped [40]. Mutual antagonistic placement of DNA methylation and H3K27me3 was also identified at the imprinted *Rasgrf1* locus in mouse [41], suggesting an evolutionary conserved basis of the underlying mechanism. Together, we conclude that DNA methylation prevents targeting of PcG proteins to sequence elements that have the potential to recruit PcG proteins.

## Materials and Methods

### Plant Material and Growth Conditions

A transgenic *Arabidopsis thaliana* (Landsberg *erecta* (L*er*)) line in which endosperm nuclei were specifically marked by EGFP was established by expressing a translational fusion of *PHE1* with EGFP under the transcriptional control of the *PHE1* promoter (*PHE1::PHE1-EGFP*) and 3 kb regulatory 3′ sequences. A transgenic Arabidopsis (Columbia, Col) line constitutively expressing YFP fused to histone H3.2 (*35S::H3.2-YFP*) served as a positive control. The *fis2-1* allele (L*er* accession) has been described previously [3]. The *met1-3* (Col accession) allele was described in [42]. For *met1; fis2* double mutant analysis the newly identified *fis2-5* allele (SALK_009910; Col accession) was used, containing a T-DNA insertion within the first exon. The *fis2-5* seed abortion ratio and mutant seed phenotypes were analyzed and found to be similar to the *fis2-1* allele (data not shown).

Seeds were surface sterilized (5% sodium hypochlorite, 0.1% Tween-20) and plated on MS medium (MS salts, 1% sucrose, pH 5.6, 0.8% bactoagar). Plants were grown in a growth cabinet under long day photoperiods (16 h light and 8 h dark) at 22°C. After 10 days, seedlings were transferred to soil and plants were grown in a growth chamber at 60% humidity and daily cycles of 16 h light at 22°C and 8 h darkness at 18°C. Inflorescences were harvested approximately 21 days after transfer to soil, shock-frozen in liquid nitrogen and stored at −80°C. For analysis of seedlings, seeds were stratified for 2 days at 4°C before incubation in a growth cabinet. After 10 days, whole seedling tissue was harvested, shock-frozen in liquid nitrogen and stored at −80°C before further usage.

### GFP Expression Analysis

Microscopy imaging was performed using a Leica DM 2500 microscope (Leica Microsystems, Wetzlar, Germany) with either bright-field or epifluorescence optics. Images were captured using a Leica DFC300 FX digital camera, exported using Leica Application Suite Version 2.4.0.R1, and processed using Photoshop 7.0 (Adobe Systems Incorporated, San Jose, USA). Confocal imaging was performed on a Leica SP1-2.

### Isolation of GFP Positive Endosperm Nuclei

Nuclei were isolated from 3.5 g of inflorescences following the protocol described in [43]. Isolated nuclei were resuspended in 1× PBS, and proteins were crosslinked to DNA with 1% formaldehyde for 8 min. After adding glycine to 125 mM final concentration and incubation for 5 min, crosslinked nuclei were washed and resuspended in 1× PBS and stained by addition of Propidium Iodide (PI) or DAPI to a final concentration of 1 µg/ml or 0.5 µg/ml, respectively. Biparametric flow analysis of EGFP fluorescence versus nuclear DNA content was performed on a fluorescence activated cell sorter (FACS Aria II, Becton, Dickinson, Franklin Lakes, USA) equipped with a 70 µm flow tip and operated at a sheath pressure of 70 psi. Events were thresholded on forward scatter and samples were sorted at the event rate of 15000/sec. For EGFP and PI excitation a 488 nm laser and for DAPI excitation a 407 laser were used. The barrier filters were 610/20 nm for PI, 450/40 for DAPI and 530/30 for EGFP fluorescence.

The position of the nuclei gate was defined using 6 µm beads (Becton Dickinson), forwards (FSC-A) and sidewards scatter (SSC-A) and was verified by DAPI-staining (Figure S4A). The position of the sort region was established by first determining the baseline of green fluorescence using inflorescence nuclei from EGFP-negative *Ler* control plants (Figure S4B). The upper and left- and right-hand boundaries of the sort window were adjusted to include all nuclei derived from YFP-positive 35S::H3.2-YFP control plants (Figure S4B). Sorted GFP positive nuclei from *PHE1::PHE1-EGFP* plants were reanalyzed to verify sorting conditions (Figure S4C).

### Transcript Level Analysis

For expression analysis from sorted nuclei, RNA was isolated by flow sorting nuclei directly into 450 µl of RLT lysis buffer (Qiagen, Hilden, Germany) and using the RNeasy Plant Mini Kit (Qiagen) according to the manufacturer's recommendation. For other expression analyses, siliques were harvested at the indicated time points and RNA extraction and generation of cDNAs were performed using RNeasy Plant Mini Kit (Qiagen) according to the supplier's instructions. For quantitative RT-PCR, RNA was treated with DNaseI and reverse transcribed using the First strand cDNA synthesis kit (Fermentas, Ontario, Canada). Gene-specific primers and Fast-SYBR-mix (Applied Biosystems, Carlsbad, USA) were used on a 7500 Fast Real-Time PCR system (Applied Biosystems). Analysis was performed using three replicates and results were analyzed as described [44]. Briefly, mean expression values and standard errors for the reference gene as well as for the target genes were determined, taking into consideration the primer efficiency that was determined for each primer pair used. Relative expression values were determined by calculating the ratio of target gene expression and reference gene expression and error bars were derived by error propagation calculation. The primers used in this study are specified in Table S7.

### Chromatin Immunoprecipitation

ChIP with 500 to 700 ng of chromatin derived from approximately 100'000 sorted nuclei was performed as described [45] using antibodies against H3K27me3 (Millipore, cat. 07-449) and rabbit IgG (Santa Cruz Biotechnology, Santa Cruz, USA, cat. Sc-2027). ChIP-DNA was amplified using the WGA-4 single cell amplification kit (Sigma-Aldrich, St. Louis, USA). For amplification of input DNA, 10 ng of chromatin was used. Amplified DNA was purified with the QIAquick PCR purification kit (Qiagen) and eluted with 50 µL of water. DNA concentration was measured using a NanoDrop 1000 (NanoDrop Technologies, Wilmington, USA).

### Microarray Analysis

#### H3K27me3 profiling

Amplified ChIP DNA was fragmented and labelled with the GeneChip WT Terminal Labeling kit (Affymetrix, Santa Clara, CA) according to the manufacturer's instructions. Fragmentation was confirmed using an RNA Nano 1000 kit on a 2100 Bioanalyzer lab-on-chip platform (Agilent, Waldbronn, Germany), revealing an average fragment size of 90 nucleotides. Labelled samples (Input, ChIP with anti-H3K27me3 and ChIP with unspecific IgG) from three independent experiments were hybridized to AGRONOMICS1 arrays (Affymetrix) as previously described [46].

#### Transcriptional profiling

The transcriptional profile of wild-type and *fis2* seeds at 3 DAP was established using ATH1 microarrays (Affymetrix) following previously published procedures [27] with three biological replicates.

#### Validation of ChIP-chip results

Selected regions were validated using independently prepared chromatin samples immunoprecipitated with H3K27me3 and IgG antibodies. Amplified ChIP-DNA was analyzed by quantitative PCR using 2 µl of 1∶30 diluted samples. Three replicates were performed for each sample and results were analyzed as described [44] and presented as percent of input. The primers used in this study are specified in Table S7.

#### Bioinformatic analysis

All analysis was performed in R 2.9.1 [47]. ChIP-chip data were normalized with MAT [48] implemented in the *aroma.affymetrix* package [49] with the window-size parameter set to 500. H3K27me3 enrichments were calculated against signals from both input and IgG samples and averaged. Enriched regions were defined as continuous runs of probes with a MAT-score of at least 3.5 and were selected using the package *BAC* [50] with minRun and maxGap parameters set to 300 and 200, respectively. A gene-specific MAT-score was defined as the 75% ile of all probe-specific MAT-scores for the probes located entirely within the transcribed region of a gene. Visualization of tiling array data was done using the Integrated Genome Browser at http://igb.bioviz.org/download.shtml [51]. Transcript profiling data were normalized with GCRMA [52]; differentially expressed genes were identified with the rankproduct algorithm [53]; false discovery rate = 0.1, fold change >0.6). Clustering analysis was performed using TM4 software [54], Enrichment of GO categories (obtained from TAIR) was tested based on the hypergeometric test and multiple-testing correction according to [55] with a critical p-value of 1.0E-03. Comparisons with whole genome data were based on the sequences probed by the AGRONOMICS1 microarray.

The transcriptional profile of wild-type and *fis2* seeds at 6 DAP has been previously published [27]. Reference transcript profiles during development were taken from [30]. DNA methylation profiles were taken from [13], [56]. Data for transcript profiles from endosperm were taken from experiments carried out in the laboratories of Bob Goldberg (UCLA), John Harada (UC Davis), Brandon Le (UCLA), Anhthu Bui (UCLA), and Julie Pelletier (UC Davis) and are available under http://estdb.biology.ucla.edu/genechip/project. Microarray raw data generated in this study are available at ArrayExpress, accession numbers E-TABM-1007 and E-TABM-1008.

## Supporting Information

Figure S1 Specific Transposon Superfamilies Are Enriched or Depleted among H3K27me3 Targets. Frequency of transposon superfamilies among endosperm-specific and shared H3K27me3 targets as well as among H3K27me3 targets in seedlings [19] in comparison to the genome-wide transposon frequency that was calculated based on sequences probed by the microarray.
(0.01 MB PDF)

Figure S2 Confirmation of Randomly Selected H3K27me3 Target Genes. A) Confirmation of endosperm-specific TEGs. B) Confirmation of endosperm-specific protein coding genes. C) Confirmation of shared H3K27me3 protein coding genes. ChIP was performed using nuclei isolated from endosperm (red bars) or seedlings (green bars) with H3K27me3 specific antibodies and randomly selected target genes were tested by qPCR. Enrichment levels are indicated as % input. Error bars correspond to standard deviation.
(0.02 MB PDF)

Figure S3 CG methylation and H3K27me3 Profiles at Selected TEGs. CG methylation profiles of TEGs in vegetative tissues and the endosperm [13], [56] were plotted together with the endosperm H3K27me3 profiles obtained in this study.
(0.49 MB PDF)

Figure S4 Establishing GFP Sorting Conditions. A) Biparametric flow sort analysis of nuclei isolated from wild-type inflorescences (upper panel), from 35S::H3.2-YFP inflorescences (middle panel) and from PHE1::PHE1-EGFP inflorescences (lower panel). P3 represents the region employed for sorting GFP-negative nuclei. P4 represents the region containing GFP-positive nuclei. B) The presence of nuclei and purity of the defined nuclei gate was verified by analyzing GFP positive nuclei isolated from PHE1:: PHE1-EGFP plants by flow cytometry before (blue line) and after DAPI staining (red line). After addition of DAPI, the whole population of particles present in the defined nuclei gate is shifted to higher DAPI fluorescence, indicating high purity of isolated nuclei. C) The purity of isolated GFP positive nuclei from PHE1::PHE1-EGFP plants was verified by re-analysis of the sorted sample. The sorted sample (green line) was clearly enriched for GFP positive nuclei compared to the unsorted sample (blue line). Bars indicate GFP positive signals. The calculated purity of nuclei was 92%. The presence of two peaks is likely contributed to endoreduplication and correspondingly increased GFP signal intensity.
(0.04 MB PDF)

Table S1 Endosperm-specific H3K27me3 targets.
(0.15 MB XLS)

Table S2 MADS-box transcription factors among shared H3K27me3 target genes.
(0.01 MB PDF)

Table S3 GO analysis of shared endosperm H3K27m3 target genes.
(0.01 MB PDF)

Table S4 Endosperm-specific H3K27me3 target genes with specific roles in cellularization and chromatin architecture.
(0.01 MB PDF)

Table S5 H3K27me3 target genes deregulated in fis2 seeds at 3 DAP and 6 DAP.
(0.01 MB PDF)

Table S6 GO analysis of genes deregulated in *fis2* at 3 DAP and 6 DAP.
(0.01 MB PDF)

Table S7 Primers used in this study.
(0.01 MB PDF)
