# Supplementary figures and images for: H3K27me3 Profiling of the Endosperm Implies Exclusion of Polycomb Group Protein Targeting by DNA Methylation
Source: PLoS Genet. 2010 Oct 7;6(10):e1001152. doi: 10.1371/journal.pgen.1001152 (PMC2951372; doi:10.1371/journal.pgen.1001152)

Figure S1

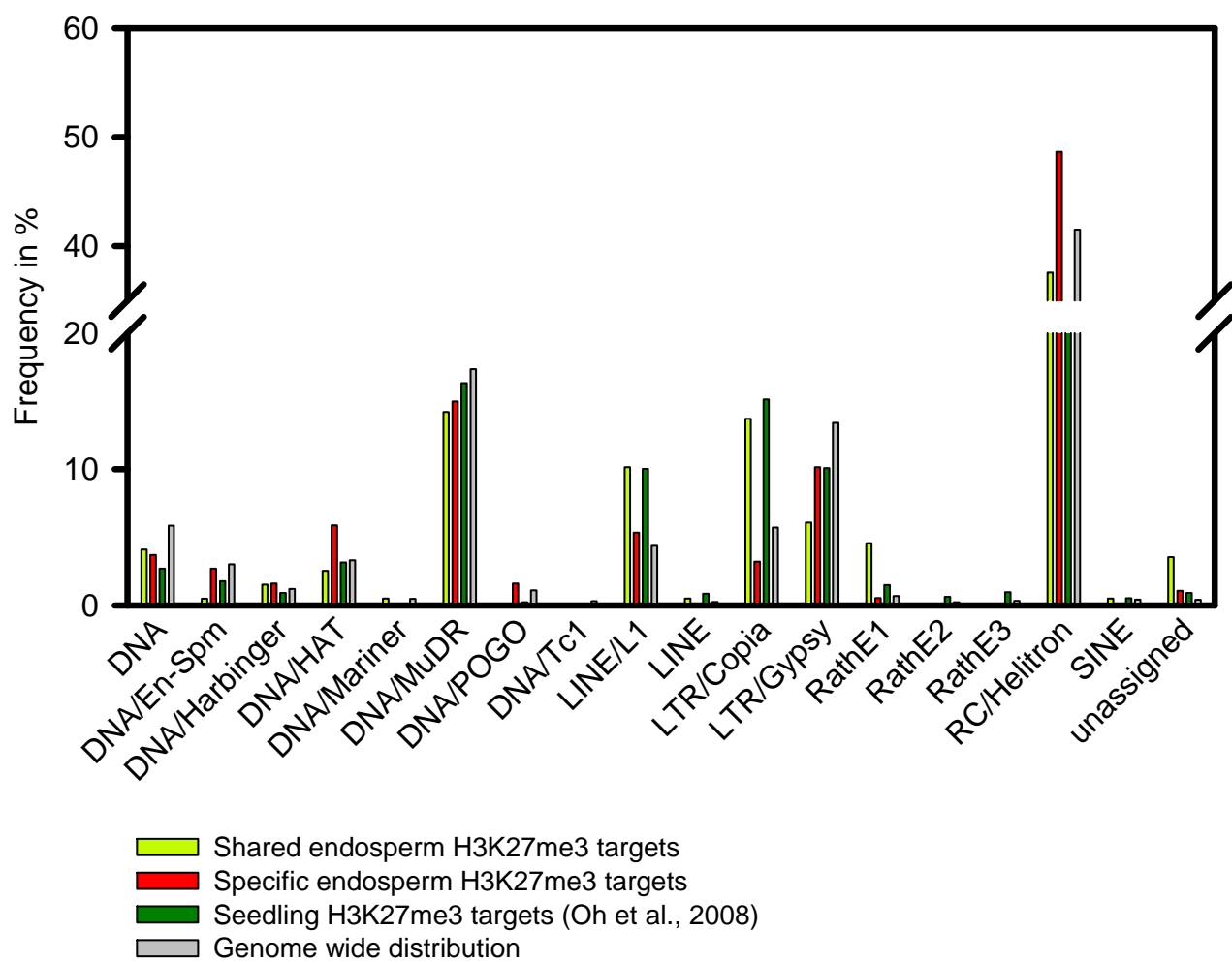

Supplement: Figure S1 — Specific Transposon Superfamilies Are Enriched or Depleted among H3K27me3 Targets. Frequency of transposon superfamilies among endosperm-specific and shared H3K27me3 targets as well as among H3K27me3 targets in seedlings [19] in comparison to the genome-wide transposon frequency that was calculated based on sequences probed by the microarray. (0.01 MB PDF) [file pgen.1001152.s001.pdf]

Figure S2

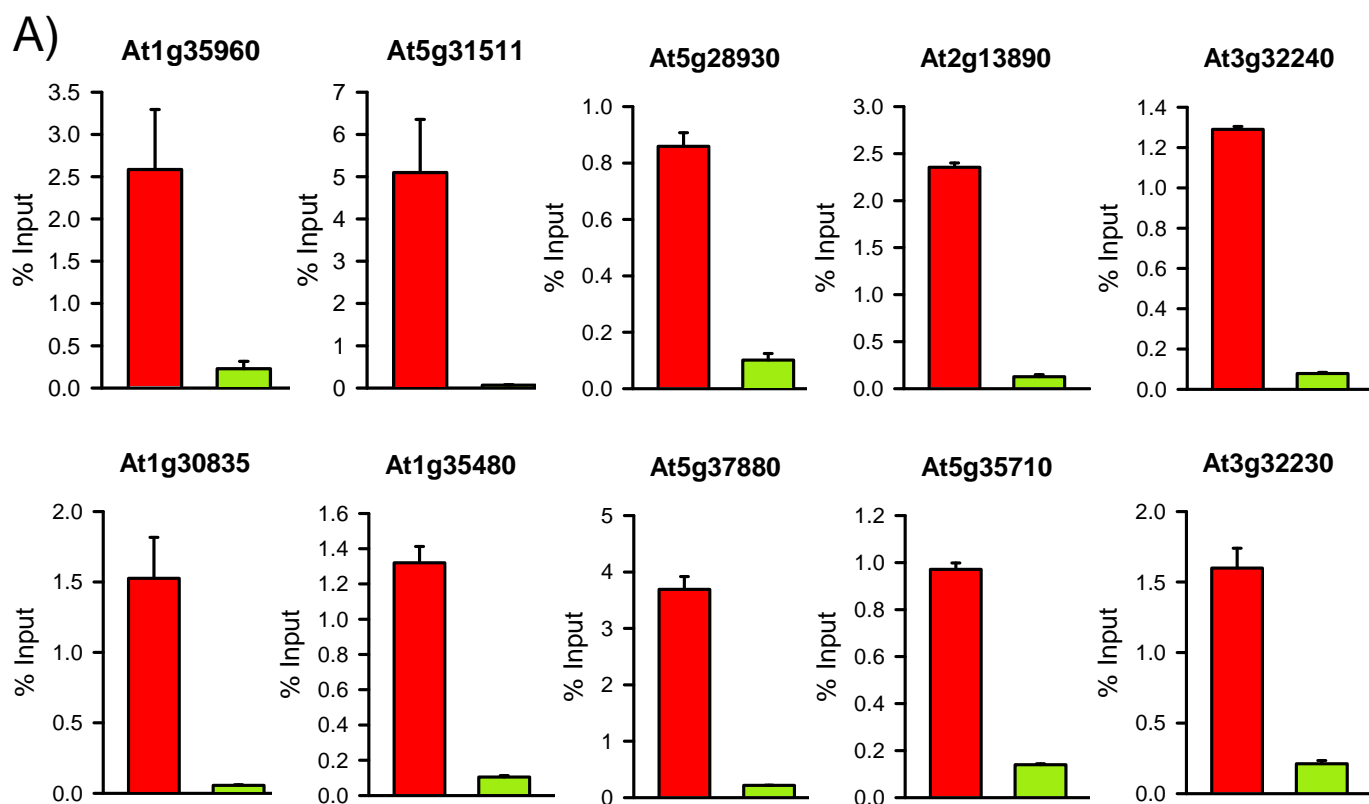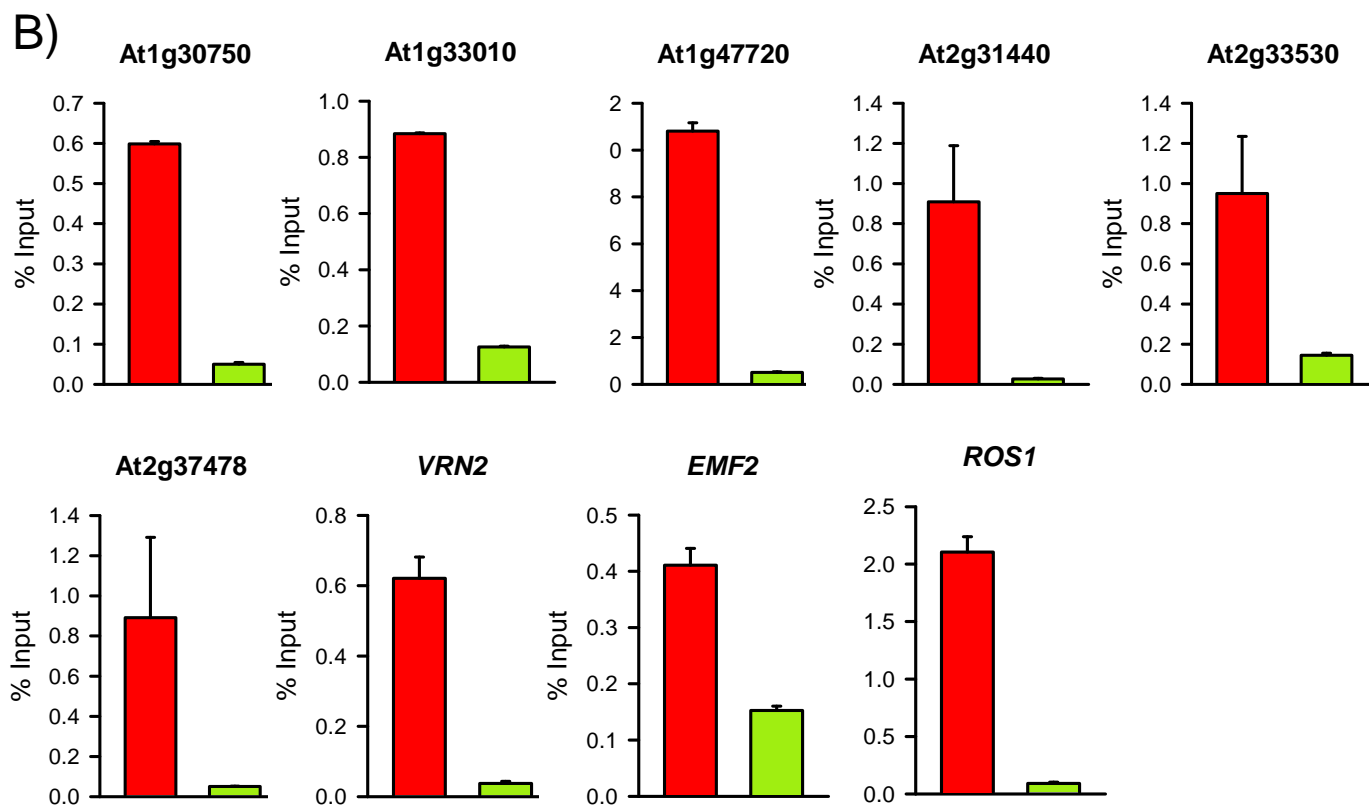

C)

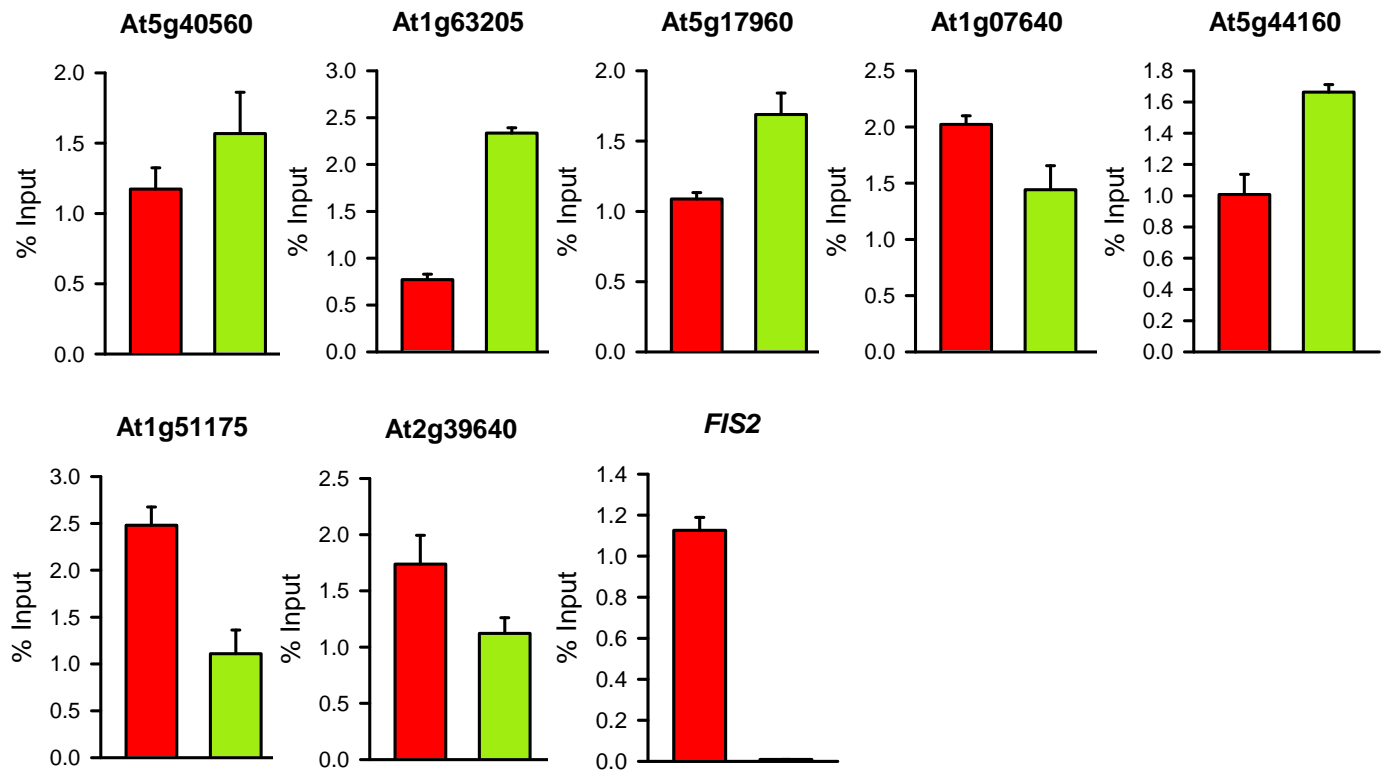

Supplement: Figure S2 — Confirmation of Randomly Selected H3K27me3 Target Genes. A) Confirmation of endosperm-specific TEGs. B) Confirmation of endosperm-specific protein coding genes. C) Confirmation of shared H3K27me3 protein coding genes. ChIP was performed using nuclei isolated from endosperm (red bars) or seedlings (green bars) with H3K27me3 specific antibodies and randomly selected target genes were tested by qPCR. Enrichment levels are indicated as % input. Error bars correspond to standard deviation. (0.02 MB PDF) [file pgen.1001152.s002.pdf]

Figure S3

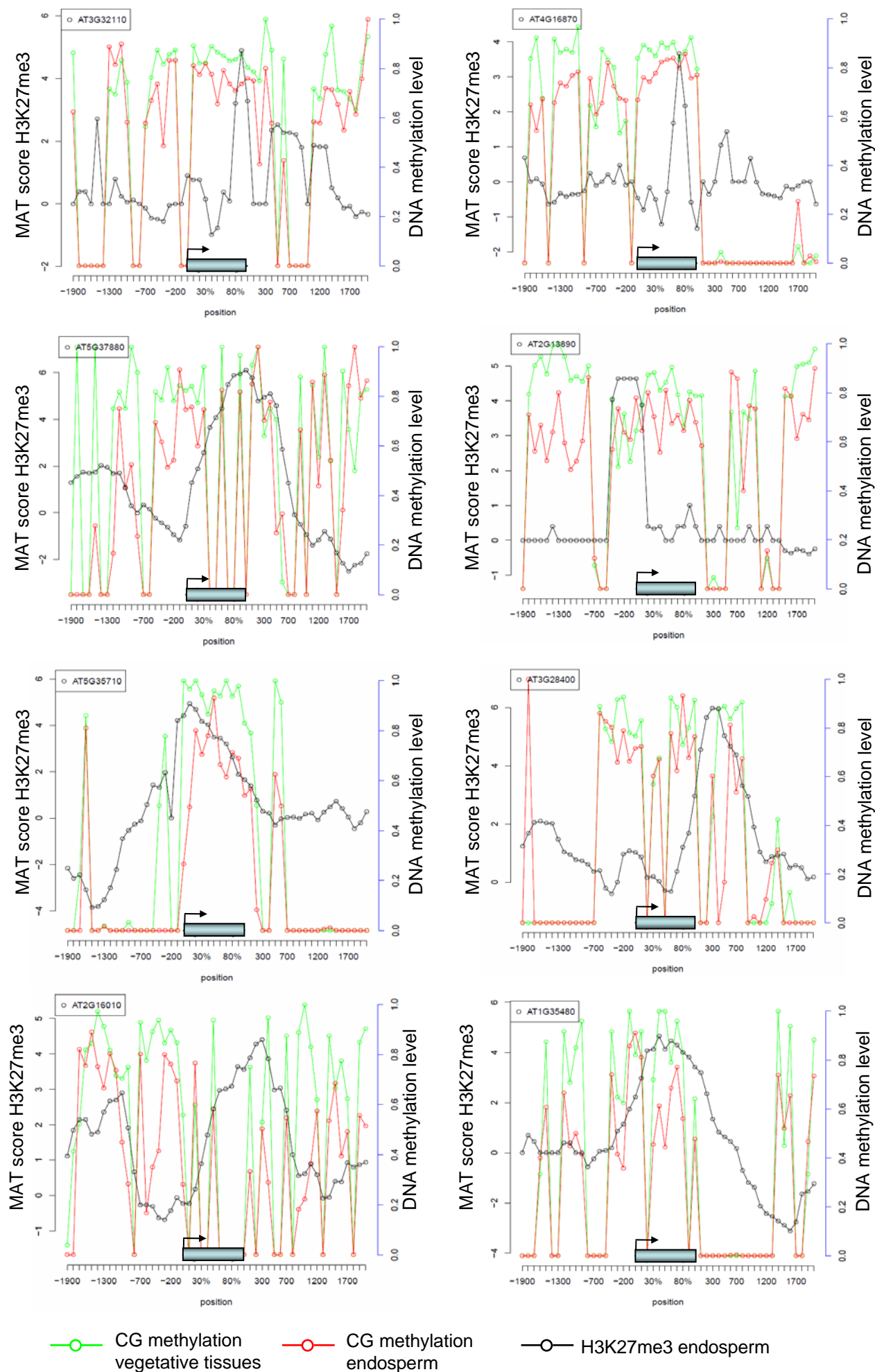

Supplement: Figure S3 — CG methylation and H3K27me3 Profiles at Selected TEGs. CG methylation profiles of TEGs in vegetative tissues and the endosperm [13], [56] were plotted together with the endosperm H3K27me3 profiles obtained in this study. (0.49 MB PDF) [file pgen.1001152.s003.pdf]

Figure S4

A)

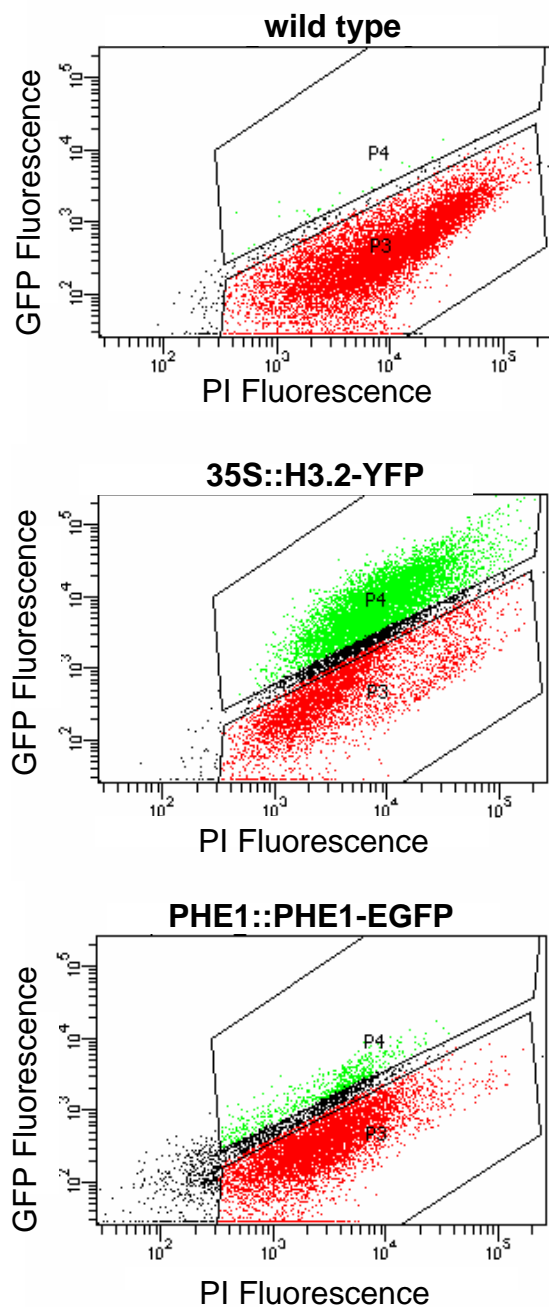

B)

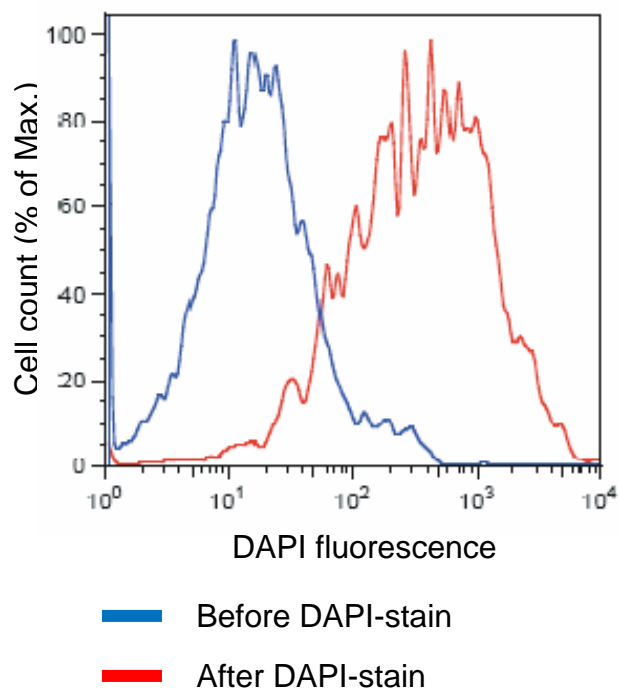

C)

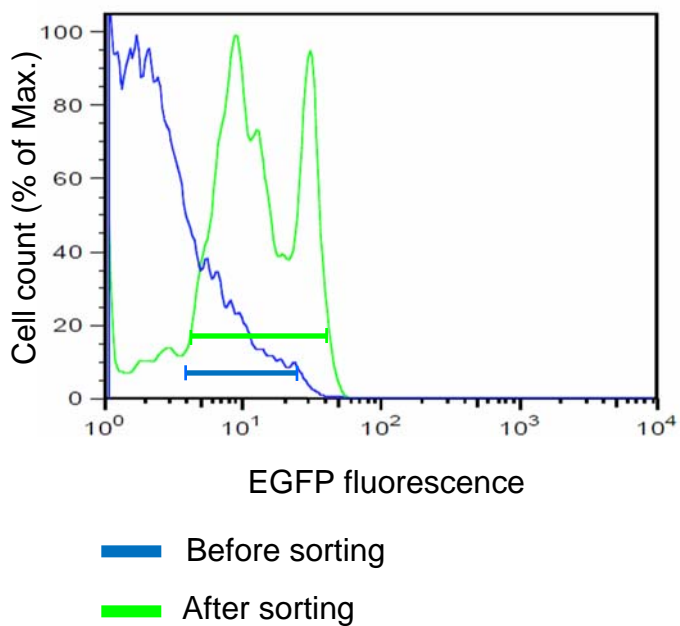

Supplement: Figure S4 — Establishing GFP Sorting Conditions. A) Biparametric flow sort analysis of nuclei isolated from wild-type inflorescences (upper panel), from 35S::H3.2-YFP inflorescences (middle panel) and from PHE1::PHE1-EGFP inflorescences (lower panel). P3 represents the region employed for sorting GFP-negative nuclei. P4 represents the region containing GFP-positive nuclei. B) The presence of nuclei and purity of the defined nuclei gate was verified by analyzing GFP positive nuclei isolated from PHE1:: PHE1-EGFP plants by flow cytometry before (blue line) and after DAPI staining (red line). After addition of DAPI, the whole population of particles present in the defined nuclei gate is shifted to higher DAPI fluorescence, indicating high purity of isolated nuclei. C) The purity of isolated GFP positive nuclei from PHE1::PHE1-EGFP plants was verified by re-analysis of the sorted sample. The sorted sample (green line) was clearly enriched for GFP positive nuclei compared to the unsorted sample (blue line). Bars indicate GFP positive signals. The calculated purity of nuclei was 92%. The presence of two peaks is likely contributed to endoreduplication and correspondingly increased GFP signal intensity. (0.04 MB PDF) [file pgen.1001152.s004.pdf]
