# Supplementary material for: H3K27me3 Profiling of the Endosperm Implies Exclusion of Polycomb Group Protein Targeting by DNA Methylation
Source: PLoS Genet. 2010 Oct 7;6(10):e1001152. doi: 10.1371/journal.pgen.1001152 (PMC2951372; doi:10.1371/journal.pgen.1001152)
Supplement: Table S2 — MADS-box transcription factors among shared H3K27me3 target genes. (0.01 MB PDF) [file pgen.1001152.s006.pdf]

**Table S2. MADS-box transcription factors among shared H3K27me3 target genes.**

| Locus | Description | Type |
|---|---|---|
| AT5G65070 | MAF4 | MIKC |
| AT3G58780 | SHATTERPROOF 1 | MIKC |
| AT1G26310 | CAULIFLOWER | MIKC |
| AT2G45650 | AGL6 | MIKC |
| AT5G60910 | AGL8 | MIKC |
| AT4G11880 | AGL14 | MIKC |
| AT5G13790 | AGL15 | MIKC |
| AT2G22630 | AGL17 | MIKC |
| AT4G37940 | AGL21 | MIKC |
| AT4G24540 | AGL24 | MIKC |
| AT5G51860 | AGL72 | MIKC |
| AT1G65330 | PHERES1 | Mγ |
| AT2G26880 | AGL41 | Mγ |
| AT5G49490 | AGL83 | Mα |
| AT1G17310 | AGL100 | Mα |
| AT3G18650 | AGL103 | Mß |
