# Supplementary material for: H3K27me3 Profiling of the Endosperm Implies Exclusion of Polycomb Group Protein Targeting by DNA Methylation
Source: PLoS Genet. 2010 Oct 7;6(10):e1001152. doi: 10.1371/journal.pgen.1001152 (PMC2951372; doi:10.1371/journal.pgen.1001152)
Supplement: Table S3 — GO analysis of shared endosperm H3K27m3 target genes. (0.01 MB PDF) [file pgen.1001152.s007.pdf]

Table S3. GO analysis of shared endosperm H3K27m3 target genes.

GO term	p-value	Number	Definition
**Functions**			
GO:0003700	1.77E-37	231	Transcription factor activity
GO:0030599	1.19E-10	22	Pectinesterase activity
GO:0016789	1.48E-09	45	Carboxylic ester hydrolase activity
GO:0005507	5.96E-09	25	Copper ion binding
GO:0045735	1.29E-07	17	Nutrient reservoir activity
GO:0016798	5.75E-06	43	Hydrolase activity, acting on glycosyl bonds
GO:0019825	4.62E-05	29	Oxygen binding
GO:0015297	8.67E-05	19	Antiporter activity
GO:0015290	8.68E-05	31	Electrochemical potential-driven transporter activity
**Processes**			
GO:0045449	6.58E-18	176	Regulation of transcription
GO:0019222	1.55E-17	182	Regulation of metabolism
GO:0019219	1.66E-17	176	Regulation of nucleobase, nucleoside, nucleotide and nucleic acid metabolism
GO:0009908	8.90E-11	37	Flower development
GO:0048569	9.28E-09	46	Post-embryonic organ development
GO:0042545	9.61E-09	23	Cell wall modification
GO:0007047	8.92E-08	32	Cell wall organization and biogenesis
GO:0045229	8.92E-08	32	External encapsulating structure organization and biogenesis
GO:0048513	1.80E-07	32	Organ development
GO:0048467	4.29E-07	13	Gynoecium development
GO:0048440	9.66E-07	12	Carpel development
GO:0007389	1.38E-06	16	Pattern specification
GO:0019748	6.20E-06	41	Secondary metabolism
GO:0050876	1.67E-05	13	Reproductive physiological process
GO:0016096	2.29E-05	16	Polyisoprenoid metabolism
GO:0006869	8.18E-05	17	Lipid transport
