# Supplementary material for: H3K27me3 Profiling of the Endosperm Implies Exclusion of Polycomb Group Protein Targeting by DNA Methylation
Source: PLoS Genet. 2010 Oct 7;6(10):e1001152. doi: 10.1371/journal.pgen.1001152 (PMC2951372; doi:10.1371/journal.pgen.1001152)
Supplement: Table S4 — Endosperm-specific H3K27me3 target genes with specific roles in cellularization and chromatin architecture. (0.01 MB PDF) [file pgen.1001152.s008.pdf]

Table S4. Endosperm-specific H3K27me3 target genes with specific roles in cellularization and chromatin architecture.

Genes regulating cellularization

Locus	Description	Function
AT3G45990	Actin-depolymerizing factor	Actin binding
AT4G29350	PROFILIN 2	Organization of actin cytoskeleton
AT4G05190	Arabidopsis thaliana kinesin 5	Microtubule motor activity
AT1G24764	Microtubule-associated proteins 70-2	Cytoskeleton organization
AT1G48760	DELTA-ADAPTIN	Vesicle-mediated transport
AT1G23900	GAMMA-ADAPTIN 1	Vesicle-mediated transport
AT1G80500	Intracellular transporter	ER to Golgi vesicle-mediated transport
AT1G60500	Dynamin family protein	Cytokinesis
AT5G35190	Proline-rich extensin-like family protein	Plant-type cell wall organization

Genes regulating chromatin architecture

AT5G51230	EMBRYONIC FLOWER 2 (EMF2)	Polycomb group protein
AT5G58230	MULTICOPY SUPRESSOR OF IRA1 (MSI1)	Polycomb group protein
AT4G16845	VERNALIZATION2 2 (VRN2)	Polycomb group protein
AT2G36490	REPRESSOR OF SILENCING 1 (ROS1)	DNA glycosylase
AT1G79890	Helicase-related	ATP-dependent DNA helicase
AT3G16600	Helicase-related	ATP-dependent DNA helicase
AT1G76110	High mobility group (HMG1/2) family protein	Chromatin architecture
AT3G13350	High mobility group (HMG1/2) family protein	Chromatin architecture
