# Supplementary material for: H3K27me3 Profiling of the Endosperm Implies Exclusion of Polycomb Group Protein Targeting by DNA Methylation
Source: PLoS Genet. 2010 Oct 7;6(10):e1001152. doi: 10.1371/journal.pgen.1001152 (PMC2951372; doi:10.1371/journal.pgen.1001152)
Supplement: Table S5 — H3K27me3 target genes deregulated in fis2 seeds at 3 DAP and 6 DAP. (0.01 MB PDF) [file pgen.1001152.s009.pdf]

Table S5. H3K27me3 target genes deregulated in *fis2* seeds at 3 DAP and 6 DAP.

H3K27me3 target genes deregulated in *fis2* seeds at 3 DAP.

Locus	Description	SLR
AT3G55130	ATWBC19 (WHITE-BROWN COMPLEX HOMOLOG 19)	0.947263
AT4G29640	Cytidine deaminase, putative	1.247031
AT4G22400	Similar to unknown protein	1.839353
AT4G20480	Similar to unknown protein	1.699762
AT4G02780	GA1 (GA REQUIRING 1)	1.227174
AT3G24250	Glycine-rich protein	0.820892
AT3G13820	F-box family protein	0.96238
AT3G10590	Myb family transcription factor	1.510954
AT1G76500	DNA-binding family protein	0.76208
AT1G76290	AMP-dependent synthetase and ligase family protein	0.794259
AT1G73000	Similar to Bet v I allergen family protein.3.	0.790477
AT1G13130	Glycosyl hydrolase family 5 protein	1.168349
AT1G23320	Alliinase family protein	0.791348
AT2G36490	ROS1 (repressor of silencing1)	1.1434
AT1G65330	PHERES1	1.305053
AT1G05280	Fringe-related protein	0.783856
AT1G62290	Aspartyl protease family protein	0.737566
AT2G25450	2-oxoglutarate-dependent dioxygenase, putative	1.082765
AT2G34700	Pollen Ole e 1 allergen and extensin family protein	1.168984
AT5G03820	GDSL-motif lipase/hydrolase family protein	1.503317

H3K27me3 target genes deregulated in *fis2* seeds at 6 DAP.

Locus	Description	SLR
AT5G06320	NDR1/HIN1-like 3	1.0846567
AT3G58780	SHATTERPROOF 1	1.1778604
AT3G50720	Protein kinase, putative	2.7418434
AT4G20800	FAD-binding domain-containing protein	3.2161362
AT4G13840	Transferase family protein	0.7156995
AT1G22015	DD46; transferase, transferring hexosyl groups	2.9658616
AT1G13680	Phospholipase C	3.0263524
AT1G58220	Myb family transcription factor	1.6323554
AT1G73610	GDSL-motif lipase/hydrolase family protein	6.5727576
AT1G47400	Similar to unknown protein	1.8830553
AT1G76290	AMP-dependent synthetase and ligase family protein	3.6689085
AT1G02940	ATGSTF5	2.7362280
AT1G75900	Family II extracellular lipase 3	2.2618593
AT1G23320	Alliinase family protein	3.7448569
AT2G24740	SET DOMAIN GROUP 21	2.9843008
AT1G17770	SET DOMAIN GROUP 21	2.9843008
AT1G65330	PHERES1	3.3128045
AT2G25700	ARABIDOPSIS SKP1-LIKE 3	1.7007573
AT1G03445	BRASSINOSTEROID INSENSITIVE 1 suppressor 1	3.6879367
AT5G39300	ARABIDOPSIS THALIANA EXPANSIN A23	8.3032823
AT3G59010	Pectinesterase family protein	1.1742109
AT2G44550	Glycosyl hydrolase family 9 protein	8.6731272
AT2G43670	Glycosyl hydrolase family protein 17	3.3372222
AT2G39640	Glycosyl hydrolase family 17 protein	2.9909098
