# Supplementary material for: H3K27me3 Profiling of the Endosperm Implies Exclusion of Polycomb Group Protein Targeting by DNA Methylation
Source: PLoS Genet. 2010 Oct 7;6(10):e1001152. doi: 10.1371/journal.pgen.1001152 (PMC2951372; doi:10.1371/journal.pgen.1001152)
Supplement: Table S6 — GO analysis of genes deregulated in fis2 at 3 DAP and 6 DAP. (0.01 MB PDF) [file pgen.1001152.s010.pdf]

Table S6. GO analysis of genes deregulated in *fis2* at 3 DAP and 6 DAP.

GO analysis of genes deregulated in *fis2* at 3 DAP.

GO term	p-value	Nr.	Definition
**Functions**			
GO:0016798	2.30E-04	9	Hydrolase activity, acting on glycosyl bonds
GO:0005372	8.01E-04	3	Water transporter activity
GO:0016667	1.16E-03	3	Oxidoreductase activity, acting on sulfur group of donors
GO:0004091	3.24E-03	4	Carboxylesterase activity
GO:0016787	3.40E-03	23	Hydrolase activity
**Processes**			
GO:0009116	2.79E-04	3	Nucleoside metabolism
GO:0009415	3.92E-04	5	Response to water
GO:0009064	9.36E-04	3	Glutamine family amino acid metabolism
GO:0009414	2.71E-03	4	Response to water deprivation
GO:0044248	4.65E-03	8	Cellular catabolism
GO:0009831	6.70E-03	2	Cell wall modification during multidimensional cell growth

GO analysis of genes deregulated in *fis2* at 6 DAP

**Functions**			
GO:0016798	5.35E-10	19	Hydrolase activity, acting on glycosyl bonds
GO:0003824	1.90E-06	88	Catalytic activity
GO:0016787	4.56E-05	38	Hydrolase activity
GO:0004091	3.60E-04	6	Carboxylesterase activity
GO:0016789	1.98E-03	8	Carboxylic ester hydrolase activity
GO:0004364	6.13E-03	3	Glutathione transferase activity
GO:0004842	6.67E-03	6	Ubiquitin-protein ligase activity
GO:0015103	7.62E-03	3	Inorganic anion transporter activity
GO:0016881	8.73E-03	6	Acid-amino acid ligase activity
GO:0008236	9.73E-03	5	Serine-type peptidase activity
**Processes**			
GO:0005975	2.85E-08	22	Carbohydrate metabolism
GO:0015698	2.61E-04	4	Inorganic anion transport
GO:0009831	8.74E-04	3	Cell wall modification during multidimensional cell growth
GO:0006820	1.05E-03	4	Anion transport
GO:0042547	1.10E-03	3	Cell wall modification during multidimensional cell growth
GO:0044248	2.41E-03	11	Cellular catabolism
GO:0009827	2.76E-03	3	Cell wall modification
GO:0005996	4.23E-03	5	Monosaccharide metabolism
GO:0009825	4.53E-03	3	Multidimensional cell growth
GO:0006355	5.11E-03	15	Regulation of transcription, DNA-dependent
GO:0009404	5.13E-03	3	Toxin metabolism
