# Supplementary material for: H3K27me3 Profiling of the Endosperm Implies Exclusion of Polycomb Group Protein Targeting by DNA Methylation
Source: PLoS Genet. 2010 Oct 7;6(10):e1001152. doi: 10.1371/journal.pgen.1001152 (PMC2951372; doi:10.1371/journal.pgen.1001152)
Supplement: Table S7 — Primers used in this study. (0.01 MB PDF) [file pgen.1001152.s011.pdf]

Table S7. Primers used in this study.

Primers for ChIP-chip validation

**Gene**	**Primer**
At1g35960	CCTCGTAAACAGAATCTCCGT
	GCATCCAGTAATTGAAACTCCA
At5g31511	CTTCTACCGAGTATCTACCTG
	ATGTTCCTTCCCATAACCTG
At5g28930	TGTTACCCACCTCTCATTACC
	CTGTTCAACTTCCCTCTCGT
At2g13890	TTCCTCACACTCCCAAATCC
	GCACAAACAAATCTCATCTCAC
At3g32240	GATTTCTTTCTAGGTTTGGCGA
	CATCTGTCTATCACTTTACTAGGG
At1g30835	CTTTAATTCCACCGATGAACCT
	CGAGAGTAGATGGTTTGTTTGAG
At1g35480	GTTGAAGTTTAGAGAAGCGT
	ATTGGATGTGGGATACTTAGAG
At5g37880	GCTTGTTGATGATAACCACAC
	TTCTTCTACACTCTCCACAATCTC
At5g35710	CTGACAACTGAATGACAAAGGG
	GAAAGGTGACGGGAGTAGAC
At3g32230	TACCGAGATTAGACCCTAACC
	TTCAAAGATGCTCCAGACAG
At1g30750	GAACTGAACCCTACACATCC
	CTTAAACTAGAACCCAAGACAG
At1g33010	TTTAGGGTTTGATCCGATTGAC
	AGGCACATTGTATCTTCCTC
At1g47720	ATGTCCCAGTTCTATTACCATCAG
	ATTCACTTCTGCCACTTTAACC
At2g31440	GCTTTACTTGTTATCACCTCTG
	CTATCATACATACATTGGCTCC

At2g33530	CACTATTGACGAGAAGAAACAG<br>TATTTAACCTCCATTGAGCCAG
At2g37478	CTCTCCTGTCTTTGATTCCT<br>TAACTAAGATTACCACGCCA
At5g40560	TGGTGGCTAATCACATATTGG<br>AACTCTTTGCTTTCGATCAC
At1g63205	TGCTATGTACACCTGAAGATCC<br>GCTCTTCGTGATTTGTTCGT
At5g17960	TGTGCACACAAACCAAACCCCCT<br>GCTTCCGACGCATTGACCAAACC
At1g07640	GCGGAGAGAGCAAGGCAGGC<br>CCAGTAGCGACGGCAACCTT
At5g44160	CCTCCACCAAGAAAAGCACACACA<br>ACTTCAGCTTCCGGATCTAAGTGGA
At1g51175	TAAGTAGTAACTGTGGATGTCGG<br>CTGTATTAATGTCGGATCACCC
At2g39640	TGGCTTCAGGTGACAACTCCACA<br>TGAATGGGGACTTAGCCTGACGA
VRN2	TAACATCTTTCACCTTCGCT<br>GCAAACTCAAACTATTAGTGGG
EMF2	CTTGATACCCGTTTCTGCTG<br>ATCCTAGAGTACAATACCATACCC
ROS1	AGAGTCAGAAATGGAGAAACAG<br>GACTGCTATGATATTGATCCTCC
FIS2	TTCAATGTTTATGGCGTGAC<br>AGAAACCATTACCACTTCCT

Primers for expression studies

BAN1	ACATTTGCTGTGCTTACAACACAAGT<br>CGAAAGCCTTCATTGATAAGTTTTTGCG
TT2	TCCTTAGAGATTACATCACCAC<br>CTTAGGTAGTTCTTCCACCGA
WOX2	CAGAACCATAAGGCTAGGCA<br>TGCTTGCTGTAAATAGTACGGA
WOX9	CGTACTATCTGATCTAGTCGTGTG<br>TACATAAACACCGTCACAACAG
At3g32110	CCTCAATTTAGACGCTCAATCC<br>AGTTCTCAAAGCCCAAACCT
At4g16870	CAATGTTGTCTCCGCTAATCC<br>CCAGATTTGAGAGGCTTTGG
At5g37880	CGCTCATGTCTAAGTCCGCT<br>ATGCTTCATCAACCCACTTG
At2g13890	AAATTCGTCAAGGGCATCAC<br>GAAACACAGATTTGGATTTGGG
At5g35710	AATTATAGGCAGCCTCCTCC<br>CGTTGTTTATCTTGGTGCGA
At1g35480	GCTCTAAGTATCCCACATCC<br>TTCAGCAACAACTCAATCCT
At3g28400	GCCTACCTTTAGATGTTAAGAC<br>TATACTGTTTATCCTCAGCCTC
At2g16010	TGTGAATGTTGGTGAAATGG<br>CTTCTTAGTGGGTTTATTGTGG
